# Undetectable = Untransmittable: A Cross-Population Systematic Review and Meta-Analysis on Awareness and Acceptance

**DOI:** 10.3390/pathogens14070673

**Published:** 2025-07-08

**Authors:** Nikolaos Georgiadis, Andreas Katsimpris, Perry N. Halkitis, Evridiki Kaba, Georgina Tzanakaki, Tonia Vassilakou, Apostolos Beloukas, Theodoros N. Sergentanis

**Affiliations:** 1 Department of Public Health Policy, School of Public Health, University of West Attica, Alexandras Avenue 196, 11521 Athens, Greece; nikgeorgiadis@uniwa.gr (N.G.); gtzanakaki@uniwa.gr (G.T.); tvasilakou@uniwa.gr (T.V.); 2 Princess Alexandra Eye Pavilion, Edinburgh EH3 9HA, UK; 3 Department of Biostatistics & Epidemiology and Center for Health Identity, Behavior & Prevention Studies, School of Public Health, Rutgers University, Newark and New Brunswick, Piscataway, NJ 08854, USA; perry.halkitis@rutgers.edu; 4 Department of Nursing, Faculty of Health and Care Sciences, University of West Attica, 12243 Athens, Greece; evridikikaba@gmail.com; 5 Department of Biomedical Sciences, University of West Attica, 12243 Athens, Greece; 6 National AIDS Reference Centre of Southern Greece, School of Public Health, University of West Attica, 12243 Athens, Greece

**Keywords:** Undetectable = Untransmittable, HIV, prevalence, infectious diseases

## Abstract

The Undetectable = Untransmittable (U=U) message is a cornerstone of HIV-related public health communication, yet global levels of awareness and acceptance remain unclear across key populations. This study aimed to assess the global prevalence of awareness and acceptance of the U=U message among men who have sex with men (MSM), people living with HIV (PLWH), healthcare professionals, and the general population. A systematic review and meta-analysis was conducted using data from PubMed, Embase, and Google Scholar without language restrictions through 31 October 2023. Eligible studies included prospective cohort studies, randomized clinical trials, and cross-sectional studies reporting numerical data on U=U awareness and acceptance. From 1171 screened records, 43 studies were included. Data were analyzed using a random effects model. The findings showed that U=U awareness was high among PLWH, moderate among MSM and healthcare professionals, and low in the general population. Complete acceptance of U=U was low in MSM and the general population, and moderate in PLWH and healthcare professionals. Any acceptance was moderate among MSM and the general population, and high among PLWH and healthcare professionals. These results highlight the need for targeted education strategies to enhance understanding and reduce HIV-related stigma, particularly in populations with lower awareness and acceptance.

## 1. Introduction

Despite international efforts, HIV persists as a pervasive global health challenge, with an estimated 1.3 million new infections and approximately 630.000 AIDS-related deaths reported globally in 2023, as documented by The Joint United Nations Programme on HIV/AIDS [1]. Sub-Saharan Africa continues to bear a disproportionate burden, accounting for roughly 65% of global new infections, while key populations—including men who have sex with men (MSM), sex workers, people who inject drugs, and transgender individuals—comprised over half of new adult infections worldwide [2]. In response to the ongoing challenges in HIV prevention, a broad range of educational strategies have been implemented to enhance awareness and reduce stigma. These include mass-media campaigns such as the global “Undetectable = Untransmittable” (U=U) initiative [3], school-based comprehensive sexuality education programs [4], and community- or peer-led interventions targeting high-risk groups [5]. More recently, digital and mHealth tools—including social media outreach, mobile apps, and SMS-based reminders—have gained traction, particularly among younger populations [6]. Among these educational initiatives, the U=U campaign, launched in 2016 by the Prevention Access Campaign, plays a pivotal role in linking effective treatment with the prevention of HIV transmission [3].

The aforementioned global initiative aims to enhance awareness regarding the efficacy of HIV treatment as prevention (TasP), highlighting that individuals living with HIV who achieve and maintain an undetectable viral load through antiretroviral therapy (ART) for at least 6 months cannot transmit the virus to their sexual partners [7,8,9]. This concept has not only transformed HIV prevention but also strengthened engagement in care and empowered PLWH to live and disclose with confidence [10]. However, the effectiveness of U=U hinges on strict ART adherence and regular viral load monitoring, and it does not prevent other sexually transmitted infections. The concept of U=U has been embedded not only in several public health initiatives, but also in clinical protocols [11], since it has been shown that patients may tend to place greater trust in information delivered by their healthcare providers [12]. Despite the fact that a wealth of scientific evidence accumulated over the past decade strongly supports U=U [8,9], awareness of this groundbreaking principal still falls significantly short of the desired level among not only PLWH but also healthcare professionals [13].

In the past, one systematic review attempted to address the awareness and other measures of U=U or TasP in different populations but included numerous studies conducted before 2016, i.e., before the conclusive establishment and dissemination of U=U, and also lacked any statistical analysis to synthesize the results [14]. This is the first systematic review and meta-analysis to date seeking to quantitatively assess the prevalence of awareness and acceptance surrounding the U=U concept across diverse populations, namely MSM, PLWH, healthcare professionals, and the general population.

## 2. Methods

### 2.1. Search Strategy

This systematic review and meta-analysis was reported according to the Preferred Reporting Items for Systematic Reviews and Meta-Analyses (PRISMA 2020) guidelines [15]; the MOOSE checklist is presented in Supplementary Table S1.

The study protocol was registered through PROSPERO (Registration No: CRD42024507710). A systematic search was conducted in the PubMed, EMBASE, and Google Scholar databases (end of search: 31 October 2023). The full search algorithms for each database are presented in the supplementary text. No language restrictions were applied. Reference lists of previously published systematic reviews and of all eligible articles were systematically searched for relevant articles in a “snowball” procedure. The results from Google Scholar were sorted by best matching and the first 1000 hits were screened.

All citations from each database were imported to a reference manager (Zotero 7.0.19) by each researcher and duplicates were removed. After initial screening of titles and abstracts, full texts of studies were evaluated. Two authors performed the selection of studies independently; disagreements were adjudicated by a senior author.

### 2.2. Eligibility

#### 2.2.1. Inclusion Criteria

We included studies focusing on MSM, PLWH, healthcare professionals, and the general population and miscellaneous population groups (i.e., studies with participants not clearly classified into the four main categories, such as HIV-negative persons at risk, people presenting for HIV testing, or minority subpopulations). Moreover, we only included studies with 10 or more participants; studies focusing on less than 10 participants were excluded.

Eligible study designs included quantitative studies, including randomized controlled trials, prospective cohorts, and cross-sectional studies, as well as qualitative studies that stated numerical data on U=U awareness and acceptance measures. Qualitative studies providing no numerical data were excluded. For conference abstracts, we thoroughly reviewed all available content, including e-posters or Supplementary Materials when accessible, to ensure that each abstract provided sufficient numerical data relevant to the outcomes assessed.

We searched for studies reporting prevalence rates or levels of awareness, familiarity, acceptance, agreement, trust, belief, or perceived accuracy related to U=U. Subsequently, we organized the gathered data into two distinct outcomes. Specifically, information derived from studies focusing on U=U awareness or familiarity was consolidated into an outcome labeled “U=U awareness.” Similarly, data from studies that assessed acceptance, agreement, perceived accuracy, trust, or belief in U=U were grouped together under “U=U acceptance”.

#### 2.2.2. Data Extraction

The following study characteristics and outcomes were extracted by both researchers and any disagreement was resolved by consulting the senior authors: title, first author, publication year, location and date of study (countries were grouped according to their income status, on the basis of World Bank data for the study publication year) [16], sample size, population characteristics (inclusion criteria), participant age (mean, median, and age range), and outcome measures. Any discrepancies in data extraction were settled by discussion and consensus with the senior authors. In the case of missing or incomplete data, the study authors were contacted via email in an effort to obtain any required information.

#### 2.2.3. Quality and Publication Bias Assessment

We assessed the risk of bias using the Newcastle–Ottawa Scale. In the analyses with 10 or more synthesized studies, publication bias was assessed with Egger’s test and by constructing a funnel plot; for the interpretation of Egger’s test, statistical significance was defined as *p* < 0.1.

#### 2.2.4. Statistical Analysis

We conducted a single analysis for U=U awareness, encompassing data regarding any level of awareness, or familiarity with U=U; individuals were considered aware if they reported any degree of familiarity, ranging from minimal awareness (“heard of U=U”) to being very familiar with the concept. On the other hand, we performed two alternative analyses for U=U acceptance. The first analysis focused on participants endorsing U=U as entirely accurate/fully agreeing (designated as “complete acceptance” throughout the manuscript). The second analysis considered any positive responses, capturing a spectrum of perceptions ranging from partial agreement or viewing U=U as somewhat accurate, to those considering it completely accurate or fully agreeing with it (designated as “any acceptance” throughout the manuscript).

Separate analyses were conducted for each outcome category within each of the five specified population groups (MSM, PLWH, healthcare professionals, the general population, and miscellaneous groups). Studies with a proportion of MSM in their total sample ≥85% were subgrouped as MSM studies.

The pooled effect estimates and 95% confidence intervals were estimated with the random effects (DerSimonian—Laird) model, using a meta-analysis of proportions with the Freeman–Tukey arcsine transformation. A three-tiered approach was adopted (0–33.3% low; 33.4–66.6% moderate; ≥66.7% high) regarding the qualitative description of the pooled proportions. Subgroup analyses by income status of the country (high; upper middle; lower middle; low), HIV status (across MSM studies) and sexual orientation (across PLWH studies) were performed. Between-study heterogeneity was assessed by estimating Q-test and I^2^ statistics. The level of statistical significance was set at 0.05.

Meta-regression analysis examining the modifying effect of the publication year, percentage of MSM (across PLWH studies), and percentage of PLWH (across MSM studies) upon the associations was performed in the analyses with ten or more synthesized studies. Statistical analysis was performed with STATA/SE version 16 (Stata Corp., College Station, TX, USA).

## 3. Results

Through our initial search, we retrieved 2390 items (560 from PubMed, 830 from Embase, and 1000 from Google Scholar). After removal of duplicates, 1171 items were screened; all details for each successive step for the selection of eligible studies are provided in the Supplementary Material (Supplementary Figure S1 and Supplementary Table S2).

Forty-three studies were included, with 227,947 participants in total, from which thirty-one were journal articles [13,17,18,19,20,21,22,23,24,25,26,27,28,29,30,31,32,33,34,35,36,37,38,39,40,41,42,43,44,45,46], and twelve were conference abstracts [47,48,49,50,51,52,53,54,55,56,57,58]. Articles included were published from 2018 to 2023 with a median sample size of 490 (interquartile range: 264–1954) ranging from 22 to 111,747. Twenty-nine studies were conducted in high-income countries [13,21,22,24,25,26,29,30,32,33,34,35,37,38,39,40,41,43,45,46,47,49,50,51,52,53,54,55,56], ten in upper-middle-income countries [18,20,23,27,28,31,36,42,44,57], three in lower-middle-income countries [17,48,58], and one study included participants from worldwide locations [19]. The study characteristics and U=U awareness and acceptance data from the included studies are summarized in Supplementary Table S3.

### 3.1. Results of the Meta-Analysis

Among the 43 included studies, 14 studies addressed U=U awareness within the MSM population [17,18,19,20,21,22,24,31,32,33,34,38,48,57], 11 provided data for the analysis on complete acceptance [17,23,27,32,33,34,35,39,44,45,49], and 12 provided data for the analysis on any acceptance within the same demographic [17,22,23,27,31,32,33,34,39,44,45,49]. Regarding PLWH, thirteen studies reported U=U awareness [13,19,21,29,31,32,33,37,38,43,47,52,57], six studies analyzed complete acceptance of U=U [13,30,33,39,44,49], whereas seven studies focused on any U=U acceptance [13,30,31,33,39,44,47]. Among healthcare professionals, six studies assessed U=U awareness [13,36,50,55,56,58], three focused on U=U complete acceptance [13,36,46], and five focused on any U=U acceptance [13,36,40,46,58]. Six studies explored U=U awareness within the general population [24,25,26,51,53,54], while one study focused on complete U=U acceptance [28] and three on any U=U acceptance within this group [28,51,53]. Finally, four studies explored miscellaneous population groups [13,41,42,44], with one focusing on HIV negative people having unprotected sex [13], another on heterosexually active Black and Latino adults [41], another on men presenting for an HIV test [42], and one on HIV negative/unknown HIV status with non-gay and non-bisexual participants [44]. Three of them reported U=U awareness [13,41,42], and two focused on complete U=U acceptance and any U=U acceptance [13,44].

**Figure 1:**
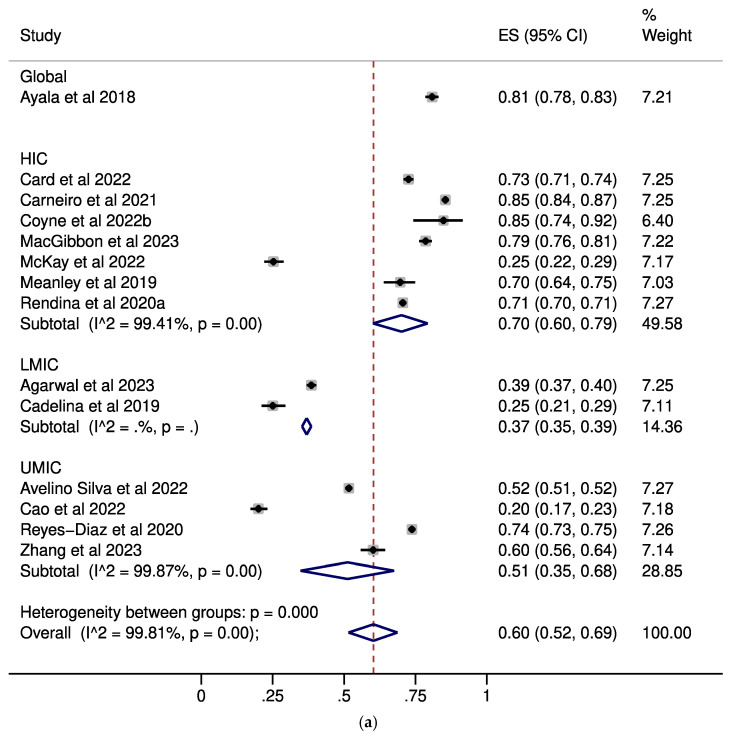

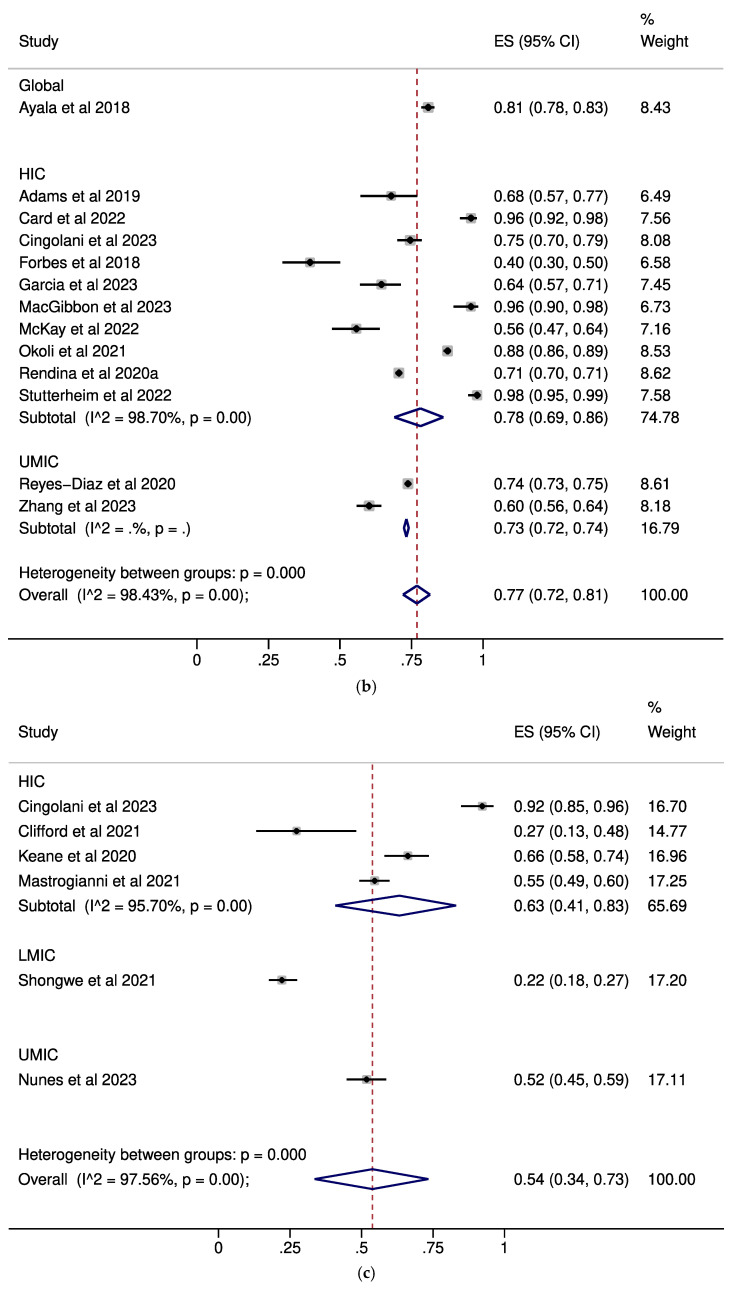

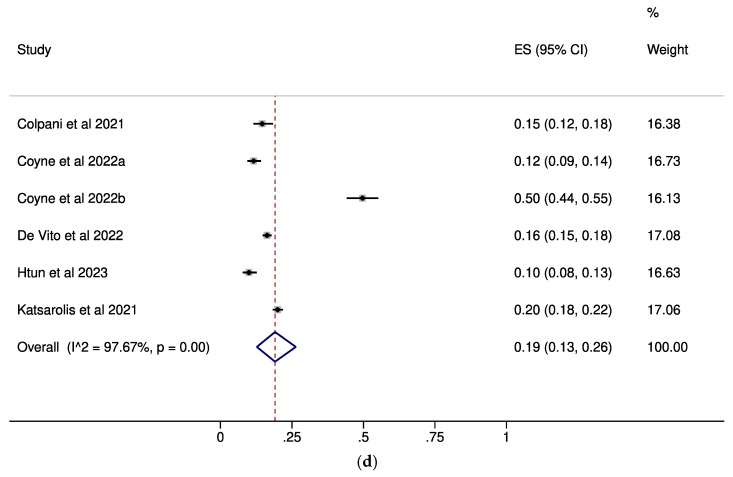
Forest plots describing the prevalence of U=U awareness in (**a**): MSM; (**b**): PLWH; (**c**): healthcare professionals; (**d**): the general population [13,17,18,19,20,21,22,24,25,26,29,31,32,33,34,36,37,38,43,47,48,50,51,52,53,54,55,56,57,58]. Subgroup analyses by the income status of the country in which each study was performed are presented. CI: confidence interval; ES: effect size; HIC: high-income countries; LMIC: low-middle-income countries; MSM: men who have sex with men; PLWH: people living with HIV; UMIC: upper-middle-income countries.

Regarding U=U awareness, moderate levels were noted among MSM (pooled ES: 0.60, 95% CI: 0.52–0.69, Figure 1a) and healthcare professionals (pooled ES: 0.54, 95% CI: 0.34–0.73, Figure 1c), while PLWH exhibited high awareness (pooled ES: 0.77, 95% CI: 0.72–0.81, Figure 1b). On the contrary, U=U awareness was low among the general population (pooled ES: 0.19, 95% CI: 0.13–0.26, Figure 1d).

**Figure 2:**
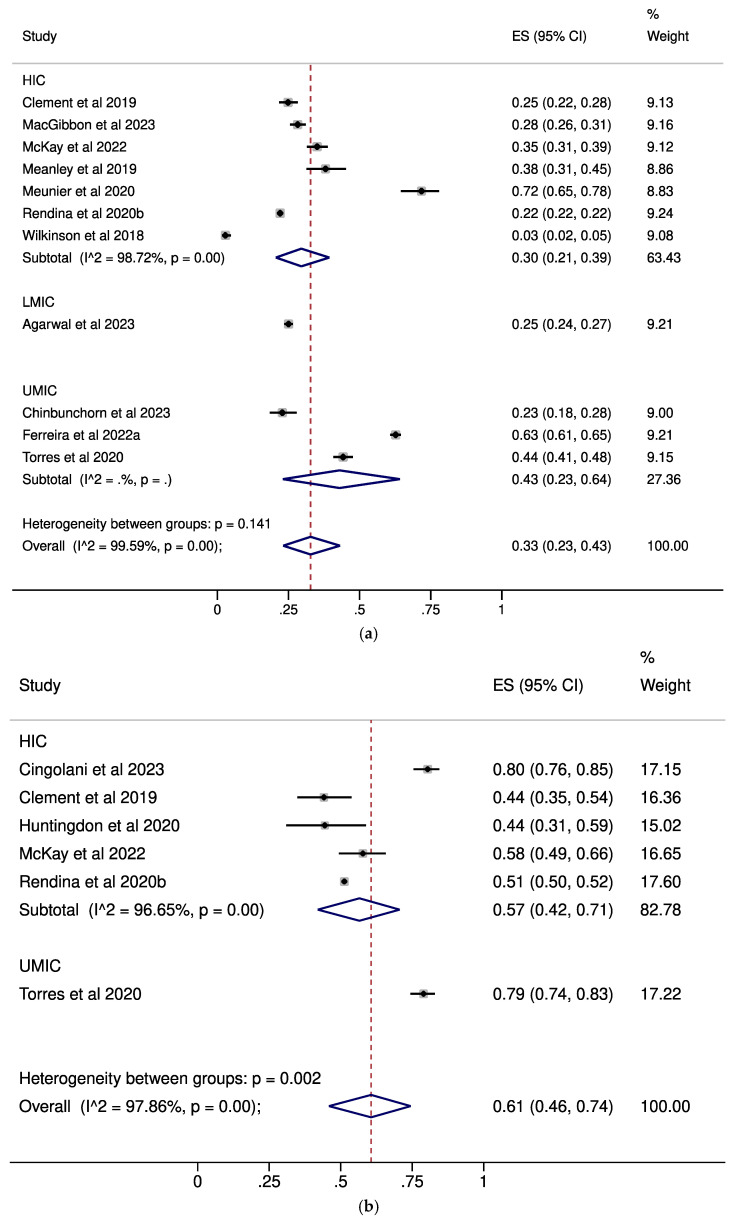

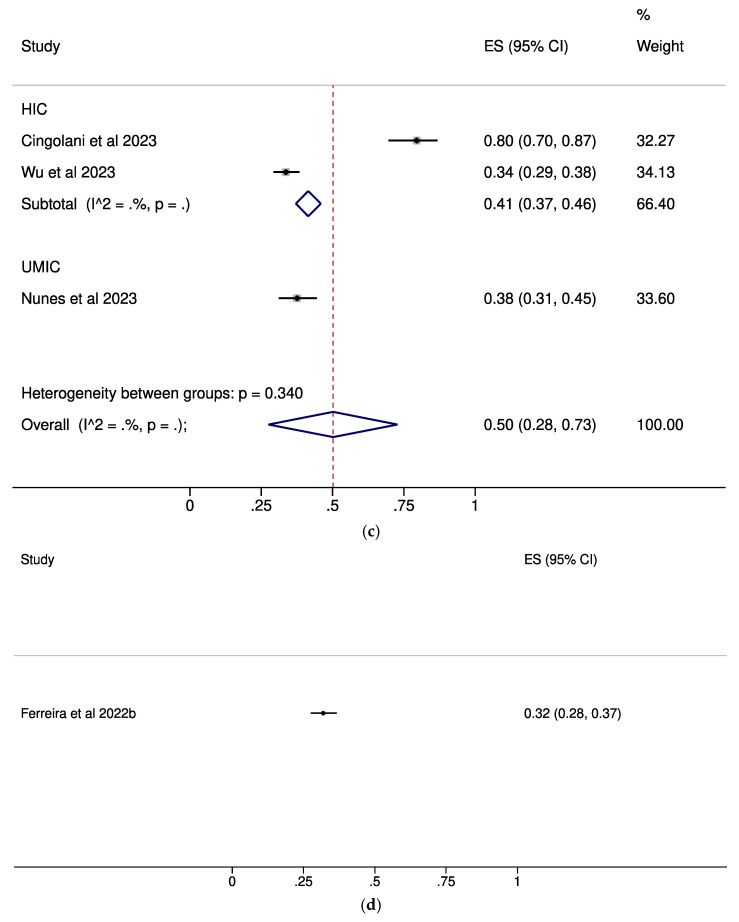
Forest plots describing the prevalence of complete U=U acceptance in (**a**): MSM; (**b**): PLWH; (**c**): healthcare professionals; (**d**): the general population [13,17,23,27,28,30,32,33,34,35,36,39,44,45,46,49]. Subgroup analyses by the income status of the country in which each study was performed are presented. CI: confidence interval; ES: effect size; HIC: high-income countries; LMIC: low-middle-income countries; MSM: men who have sex with men; PLWH: people living with HIV; UMIC: upper-middle-income countries.

In regard to complete acceptance of U=U, low levels were noted in MSM (pooled ES: 0.33, 95% CI: 0.23–0.43, Figure 2a) and the general population (pooled ES: 0.32, 95% CI: 0.28–0.37, Figure 2d), whereas moderate levels were demonstrated by PLWH (pooled ES: 0.61, 95% CI: 0.46–0.74, Figure 2b) and healthcare professionals (pooled ES: 0.50, 95% CI: 0.28–0.73, Figure 2c).

**Figure 3:**
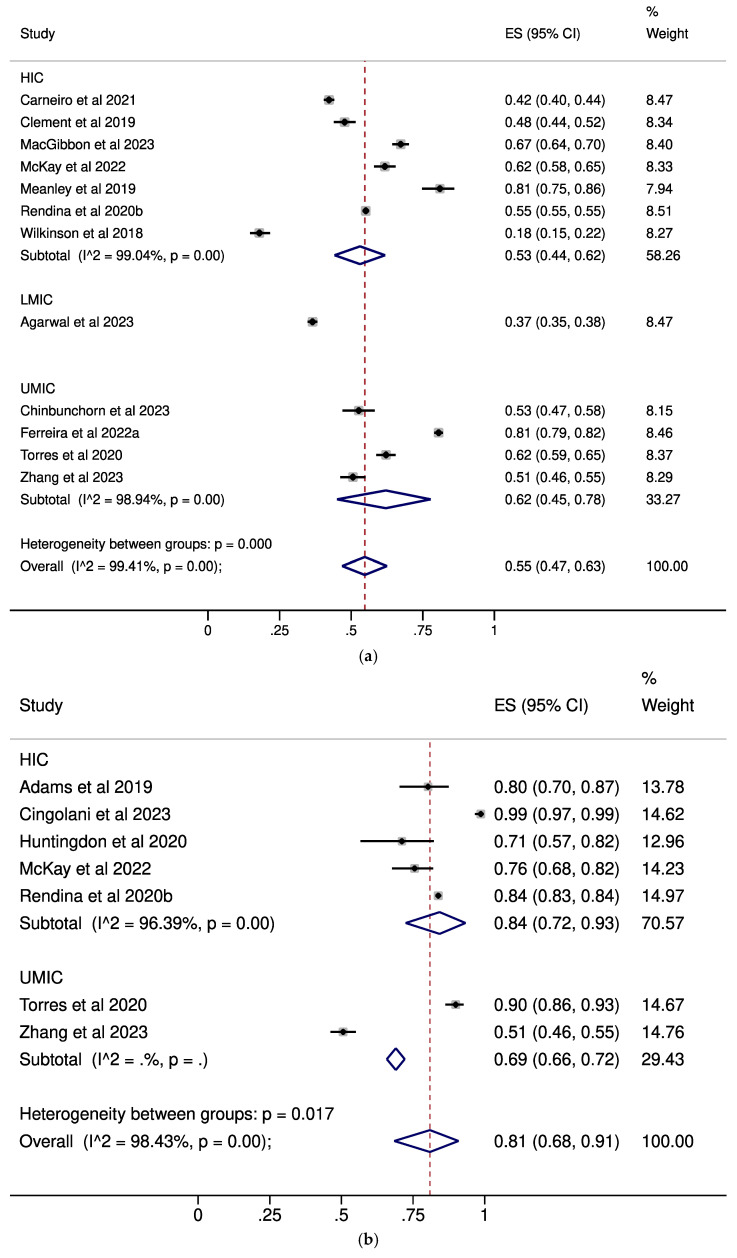

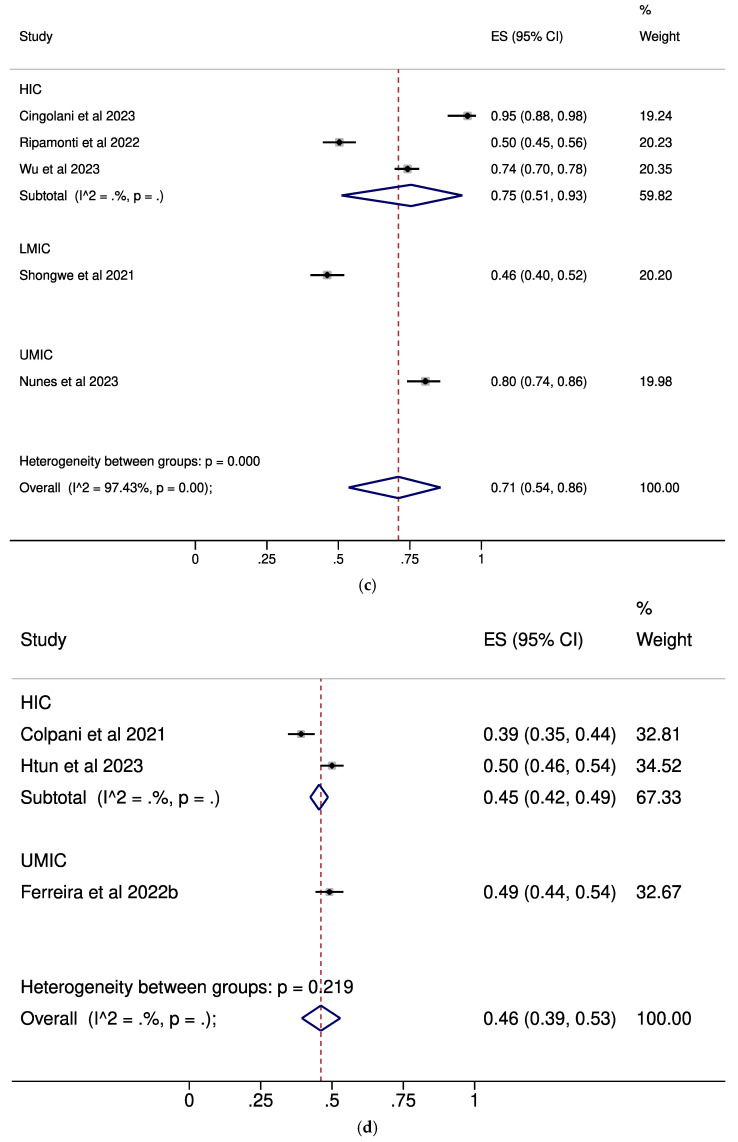
Forest plots describing the prevalence of any U=U acceptance in (**a**): MSM; (**b**): PLWH; (**c**): healthcare professionals; (**d**): the general population [13,17,22,23,27,28,30,31,32,33,34,36,39,40,44,45,46,47,49,51,53,58]. Subgroup analyses by the income status of the country in which each study was performed are presented. CI: confidence interval; ES: effect size; HIC: high-income countries; LMIC: low-middle-income countries; MSM: men who have sex with men; PLWH: people living with HIV; UMIC: upper-middle-income countries.

Moreover, concerning any acceptance of U=U, moderate levels were exhibited by MSM (pooled ES: 0.55, 95% CI: 0.47–0.63, Figure 3a) and the general population (pooled ES: 0.46, 95% CI: 0.39–0.53, Figure 3d), while high levels were observed in PLWH (pooled ES: 0.81, 95% CI: 0.68–0.91, Figure 3b) and healthcare professionals (pooled ES: 0.71, 95% CI: 0.54–0.86, Figure 3c). All results, including subgroup analyses by income status along with results for miscellaneous categories, are presented in Table 1. The forest plots for the subgroup analyses based on the percentage of PLWH among MSM are depicted in Supplementary Figures S2–S4, while the subgroup analyses focusing on sexual orientation within the PLWH category are shown in Supplementary Figures S5–S7. Additional analyses covering miscellaneous categories are presented in Supplementary Figures S8–S10.

As a rule, the results were replicated within the high-income-country subgroup, which represented the majority of studies. Additionally, replication was observed within the HIV-negative/unknown subgroup for MSM studies, as well as within the MSM subgroup for PLWH studies, both of which constituted the majority of studies within their respective categories. The results of the meta-regression analysis are shown in Supplementary Table S4. No significant modifying effects of publication year, PLWH percentage, or sexual orientation upon the studied outcomes were documented.

### 3.2. Evaluation of Quality of Studies and Risk of Bias

The evaluation of the quality of the included studies is presented in Supplementary Table S5. The extraction of unadjusted data and the inability for a blind assessment of the outcomes compromised the overall quality of all the included studies. No significant publication bias was detected via Egger’s test in the analysis of U=U awareness in MSM (*p* = 0.553), U=U awareness in PLWH (*p* = 0.357), complete U=U acceptance in MSM (*p* = 0.158), or any U=U acceptance in MSM (*p* = 0.900) (Supplementary Figures S11–S14).

## 4. Discussion

This systematic review and meta-analysis unveiled varying levels of U=U awareness across different demographic groups. Among MSM and healthcare professionals, awareness levels were moderate, while PLWH exhibited high levels of awareness. Conversely, U=U awareness was low among the general population. When examining complete acceptance of U=U, MSM and the general population demonstrated low levels, whereas PLWH and healthcare professionals displayed moderate levels. Furthermore, regarding any acceptance of U=U, moderate levels were observed among MSM and the general population, while PLWH and healthcare professionals exhibited high levels.

The observed disparity in levels of U=U awareness and acceptance between PLWH and MSM is reinforced by evidence that individuals actively involved in HIV treatment and prevention and thus in regular contact with healthcare professionals are more likely to be aware and accept U=U [59]. Additionally, further gaps in U=U messaging among MSM may contribute to these differences [22]. The notable yet imperfect levels of U=U awareness and acceptance among healthcare professionals suggest a potential knowledge gap stemming from inadequate education during medical training [60]. This educational shortfall not only affects awareness and acceptance levels but also fosters uncertainty regarding the unequivocal accuracy of U=U messaging [61]. Lower income and education levels have been identified as barriers to U=U awareness and acceptance within the general population, alongside other potential factors, such as inadequate sexual health education, that have yet to be quantified in research studies [28]. Understanding the points at which to intervene is crucial to diminish the gaps in the HIV care continuum, especially among high-risk groups like young MSM, among others [62].

Tailored strategies are needed to improve U=U knowledge among populations with limited awareness, such as the general public and MSM in low- and middle-income countries. Targeted mass-media campaigns using clear, culturally relevant messaging can help normalize U=U across broader audienc es [63]. Additionally, community- and peer-led interventions that integrate U=U education into HIV testing and PrEP services may effectively reach MSM and other key populations. In clinical settings, training healthcare professionals to proactively discuss U=U with patients—especially those newly diagnosed or at risk—can also play a pivotal role [60]. Incorporating U=U messages into school-based sexuality education and digital platforms (e.g., social media, mobile apps) could further enhance outreach, particularly among youth and hard-to-reach groups.

Several limitations should be considered when interpreting the results of the present systematic review and meta-analysis. First, the included studies exhibited significant variability in study design, geographical location, and participant demographics. Second, our meta-analysis primarily drew upon data from high-income countries, thereby limiting the generalizability of findings to other regions. This underscores the necessity for additional studies in diverse socioeconomic contexts to foster a more comprehensive understanding. Third, potential participation bias may have influenced results, as individuals with greater familiarity or stronger opinions regarding U=U might have been more likely to participate, potentially skewing awareness and acceptance levels. Additionally, there may be variability across studies in how “complete acceptance” and “any acceptance” of U=U were defined and measured, which could influence the comparability of pooled estimates. Moreover, publication date may have influenced our findings, as awareness and acceptance of U=U likely evolved during the study period, potentially introducing chronological bias across included studies. Last, the diverse range of outcome measures used across studies to assess U=U awareness and acceptance represents another limitation of this systematic review.

On the other hand, our study demonstrates several strengths. One significant advantage is its pioneering nature as the first meta-analysis quantitatively assessing U=U awareness and acceptance. Additionally, we included a diverse range of populations, such as MSM, PLWH, healthcare professionals, and the general population, ensuring a comprehensive evaluation of U=U awareness and acceptance across various demographics. Furthermore, with a total of 227,947 participants across 43 studies, our study benefits from a substantial sample size, enhancing the statistical power and precision of the synthesized results.

In summary, our research illuminates the present states of awareness and acceptance of U=U among diverse populations, but it also emphasizes the continuous need for coordinated initiatives to spread this vital knowledge. Through tackling inequalities in awareness and acceptance, we can work towards alleviating the stigma associated with HIV and hence create a more inclusive and equitable world for people living with HIV. Continued advocacy and education initiatives are essential to ensure that accurate information about U=U reaches all individuals.

## Figures and Tables

**Table 1:** Results of the meta-analyses examining the prevalence of U=U awareness, complete U=U acceptance, and any U=U acceptance in MSM, PLWH, healthcare professionals, and the general population and miscellaneous groups; subgroup analyses by income status, PLWH percentage, and sexual orientation are presented.

		Prevalence Rates	
	n	ES (95%CI:)	Heterogeneity I^2^, *p*
**MSM**			
**U=U awareness**			
Overall Analysis	14	0.60 (0.52–0.69)	99.81%, *p* < 0.001
Subgroups by Income Status			
HIC	7	0.70 (0.60–0.69)	99.41%, *p* < 0.001
UMIC	4	0.51 (0.35–0.68)	NC
LMIC	2	0.37 (0.35–0.39)	NC
Global	1	0.81 (0.78–0.83)	NC
Subgroup by PLWH Percentage			
Any HIV Status	5	0.54 (0.40–0.67)	99.70%, *p* < 0.001
HIV-Negative/Unknown	3	0.59 (0.14–0.96)	NC
PLWH	4	0.72 (0.68–0.76)	97.32%, *p* < 0.001
Not Reported	2	0.33 (0.9–0.37)	NC
**U=U complete acceptance**			
Overall Analysis	12	0.33 (0.23–0.43)	99.59%, *p* < 0.001
Subgroups by Income Status			
HIC	8	0.30 (0.21–0.39)	98.72%, *p* < 0.001
UMIC	3	0.43 (0.23–0.64)	NC
LMIC	1	0.25 (0.24–0.27)	NC
Subgroups by PLWH percentage			
Any HIV Status	7	0.31 (0.20–0.43)	99.68%, *p* < 0.001
HIV-Negative/Unknown	4	0.36 (0.09–0.69)	99.41%, *p* < 0.001
**U=U any acceptance**			
Overall Analysis	12	0.55 (0.47–0.63)	99.46%, *p* < 0.001
Subgroups by Income Status			
HIC	7	0.53 (0.44–0.62)	99.04%, *p* < 0.001
UMIC	4	0.62 (0.45–0.78)	NC
LMIC	1	0.37 (0.35–0.38)	NC
Subgroups by PLWH percentage			
Any HIV Status	7	0.58 (0.47–0.68)	99.54%, *p* < 0.001
HIV-Negative/Unknown	4	0.51 (0.31–0.70)	99.2%, *p* < 0.001
PLWH	1	0.51 (0.46–0.63)	NC
**PLWH**			
**U=U awareness**			
Overall Analysis	13	0.77 (0.72–0.81)	98.43%, *p* < 0.001
Subgroups by Income Status			
HIC	10	0.78 (0.69–0.86)	98.70%, *p* < 0.001
UMIC	2	0.73 (0.72–0.74)	NC
Global	1	0.81 (0.78–0.83)	NC
Subgroups by Sexual Orientation			
MSM	7	0.77 (0.73–0.81)	97.71%, *p*<.001
MSM and Heterosexual	3	0.88 (0.76–0.97)	NC
MSM and Heterosexual	3	0.88 (0.76–0.97)	NC
Heterosexual	1	0.40 (0.30–0.50)	NC
Not Reported	2	0.66 (0.60–0.71)	NC
**U=U complete acceptance**			
Overall Analysis	6	0.61 (0.46–0.74)	97.86%, *p* < 0.001
Subgroups by Income Status			
HIC	5	0.57 (0.42–0.83)	96.65%, *p* < 0.001
UMIC	1	0.79 (0.74–0.83)	NC
Subgroups by Sexual Orientation			
MSM	3	0.51 (0.46–0.57)	NC
MSM and Heterosexual	3	0.71 (0.57–0.83)	NC
**U=U any acceptance**			
Overall Analysis	7	0.81 (0.68–0.91)	98.43%, *p* < 0.001
Subgroups by Income Status			
HIC	5	0.84 (0.72–0.93)	96.39%, *p* < 0.001
UMIC	2	0.69 (0.66–0.72)	NC
Subgroups by Sexual Orientation			
MSM	3	0.71 (0.45–0.91)	NC
MSM and Heterosexual	3	0.90 (0.75–0.99)	NC
Not Reported	1	0.80 (0.70–0.87)	NC
**Healthcare Professionals**			
**U=U awareness**			
Overall Analysis	6	0.54 (0.34–0.73)	97.56%, *p* < 0.001
Subgroups by Income Status			
HIC	4	0.63 (0.41–0.83)	95.7%, *p* < 0.001
UMIC	1	0.52 (0.45–0.59)	NC
LMIC	1	0.22 (0.18–0.27)	NC
**U=U complete acceptance**			
Overall Analysis	3	0.50 (0.28–0.73)	NC
Subgroups by Income Status			
HIC	2	0.41 (0.37–0.46)	NC
UMIC	1	0.38 (0.31–0.45)	NC
**U=U any acceptance**			
Overall Analysis	5	0.71 (0.54–0.86)	97.43%, *p* < 0.001
Subgroups by Income Status			
HIC	3	0.75 (0.51–0.93)	NC
UMIC	1	0.80 (0.74–0.86)	NC
LMIC	1	0.46 (0.40–0.52)	NC
**General Population**			
**U=U awareness**			
Overall Analysis	6	0.19 (0.13–0.26)	97.67%, *p* < 0.001
**U=U complete acceptance**			
Overall Analysis	1	0.32 (0.28–0.37)	NC
**U=U any acceptance**			
Overall Analysis	3	0.46 (0.39–0.53)	NC
Subgroups by Income Status			
HIC	2	0.45 (0.42–0.49)	NC
UMIC	1	0.49 (0.44–0.54)	NC
**Miscellaneous groups**			
**U=U awareness**			
HIV-Negative People Having Unprotected Sex	1	0.47 (0.43–0.51)	NC
Heterosexual Black and Latino Adults	1	0.35 (0.31–0.39)	NC
Men Presenting for An HIV Test	1	0.70 (0.64–0.76)	NC
HIV-Negative People Having Unprotected Sex	1	0.67 (0.62–0.72)	NC
HIV-Negative/Unknown HIV Status, Non-Gay Non-Bisexual	1	0.17 (0.14–0.21)	NC
**U=U any acceptance**			
HIV-Negative People Having Unprotected Sex	1	0.98 (0.95–0.99)	NC
HIV-Negative/Unknown HIV Status, Non-Gay Non-Bisexual	1	0.31 (0.27–0.35)	NC

CI: confidence interval; ES: effect size; HIC: high-income countries; LMIC: low-middle-income countries; MSM: men who have sex with men; NC: not calculated; PLWH: people living with HIV; UMIC: upper-middle-income countries.

## Data Availability

All data from each included study are accessible to anyone reading the manuscript in Supplementary Table S3 of the Supplementary Material.

## Supplementary Materials

The following supporting information can be downloaded at: https://www.mdpi.com/article/10.3390/pathogens14070673/s1. Figure S1. PRISMA 2020 flow chart; Figure S2. Forest plot describing the prevalence of U=U awareness in MSM. Subgroup analyses by the percentage of PLWH is presented; Figure S3. Forest plot describing the prevalence of complete U=U acceptance in MSM. Subgroup analyses by the percentage of PLWH is presented; Figure S4. Forest plot describing the prevalence of any U=U acceptance in MSM. Subgroup analyses by the percentage of PLWH is presented; Figure S5. Forest plot describing the prevalence of U=U awareness in PLWH. Subgroup analyses by sexual orientation is presented; Figure S6: Forest plot describing the prevalence of complete U=U acceptance in PLWH. Subgroup analyses by sexual orientation is presented; Figure S7: Forest plot describing the prevalence of any U=U acceptance in PLWH. Subgroup analyses by sexual orientation is presented; Figure S8. Forest plot describing the prevalence of U=U awareness prevalence in miscellaneous categories; Figure S9. Forest plot describing the prevalence of complete U=U acceptance in miscellaneous categories; Figure S10. Forest plot describing the prevalence of any U=U acceptance in miscellaneous categories; Figure S11. Funnel plot of the meta-analysis on U=U awareness in MSM; Figure S12. Funnel plot of the meta-analysis on complete U=U acceptance in MSM; Figure S13. Funnel plot of the meta-analysis on any U=U acceptance in MSM; Figure S14. Funnel plot of the meta-analysis on U=U awareness in PLWH; Table S1: PRISMA Checklist; Table S2: Studies excluded with their reason for exclusion; Table S3. U=U awareness and acceptance data in eligible studies; Table S4: Meta-regression analysis examining the role of publication year as a potential modifier on U=U awareness, complete acceptance and any acceptance in MSM and U=U awareness in PLWH; Table S5: Evaluation of the eligible studies with Newcastle-Ottawa scale.
