# Supplementary material for: Undetectable = Untransmittable: A Cross-Population Systematic Review and Meta-Analysis on Awareness and Acceptance
Source: Pathogens. 2025 Jul 8;14(7):673. doi: 10.3390/pathogens14070673 (PMC12298288; doi:10.3390/pathogens14070673)
Supplement: Supplementary file 1 [file pathogens-14-00673-s001.zip › pathogens-3707928-supplementary.pdf]

Supplemental Material

Abbreviations: Page 2

Supplemental Tables: Pages 3-14

Supplemental Table S1: PRISMA Checklist: Pages 3-4

Supplemental Table S2: Studies excluded with their reason for exclusion: Pages 5-6

Supplemental Table S3. U=U awareness and acceptance data in eligible studies: Pages 7-13

Supplemental Table S4: Meta-regression analysis examining the role of publication year as a potential modifier on U=U awareness, complete acceptance and any acceptance in MSM and U=U awareness in PLWH: Page 14

Supplemental Table S5: Evaluation of the eligible studies with Newcastle-Ottawa scale: Pages 15-18

Supplemental Figures: Pages 19-32

Supplemental Figure S1. PRISMA 2020 flow chart: Page 19

Supplemental Figure S2. Forest plot describing the prevalence of U=U awareness in MSM. Subgroup analyses by the percentage of PLWH is presented: Page 20

Supplemental Figure S3. Forest plot describing the prevalence of complete U=U acceptance in MSM. Subgroup analyses by the percentage of PLWH is presented: Page 21

Supplemental Figure S4. Forest plot describing the prevalence of any U=U acceptance in MSM. Subgroup analyses by the percentage of PLWH is presented: Page 22

Supplemental Figure S5. Forest plot describing the prevalence of U=U awareness in PLWH. Subgroup analyses by sexual orientation is presented: Page 23

Supplemental Figure S6: Forest plot describing the prevalence of complete U=U acceptance in PLWH. Subgroup analyses by sexual orientation is presented: Page 24

Supplemental Figure S7: Forest plot describing the prevalence of any U=U acceptance in PLWH. Subgroup analyses by sexual orientation is presented: Page 25

Supplemental Figure S8. Forest plot describing the prevalence of U=U awareness prevalence in miscellaneous categories: Page 26

Supplemental Figure S9. Forest plot describing the prevalence of complete U=U acceptance in miscellaneous categories: Page 27

Supplemental Figure S10. Forest plot describing the prevalence of any U=U acceptance in miscellaneous categories: Page 28

Supplemental Figure S11. Funnel plot of the meta-analysis on U=U awareness in MSM: Page 29

Supplemental Figure S12. Funnel plot of the meta-analysis on complete U=U acceptance in MSM: Page 30

Supplemental Figure S13. Funnel plot of the meta-analysis on any U=U acceptance in MSM: Page 31

Supplemental Figure S14. Funnel plot of the meta-analysis on U=U awareness in PLWH: Page 32

Supplemental Text: Search algorithm for PubMed, Scopus and Embase: Page 33

References: Page 34-38

Abbreviations: AIDS: Acquired immunodeficiency syndrome, ART: antiretroviral therapy, CI: Confidence Interval, ES: Effect size, HIC: High Income Countries HIV: Human immunodeficiency virus, LIC: Low Income Countries, LMIC: Lower Middle Income Countries MSM: Men who have sex with men, NOS: Newcastle Ottawa Scale, PLWH: People Living with HIV, PrEP: Pre-Exposure Prophylaxis, PR: Prevalence Ratio, PRISMA: Preferred Reporting Items for Systematic Reviews and Meta-Analyses, STI: Sexually Transmitted Infection, TM: Transgender Men, TASP: treatment as prevention, TW: Transgender Women, UMIC, Upper Middle Income Countries, UNAIDS: United Nations Program on HIV/AIDS, U=U: Undetectable = Untransmittable, WHO: World Health Organization

Prisma Checklist 2020

Section and Topic	Item #	Checklist item	Location where item is reported
**TITLE**			
Title	1	Identify the report as a systematic review.	4
**ABSTRACT**			
Abstract	2	See the PRISMA 2020 for Abstracts checklist.	2-3
**INTRODUCTION**			
Rationale	3	Describe the rationale for the review in the context of existing knowledge.	4
Objectives	4	Provide an explicit statement of the objective(s) or question(s) the review addresses.	4
**METHODS**			
Eligibility criteria	5	Specify the inclusion and exclusion criteria for the review and how studies were grouped for the syntheses.	5-6
Information sources	6	Specify all databases, registers, websites, organisations, reference lists and other sources searched or consulted to identify studies. Specify the date when each source was last searched or consulted.	5
Search strategy	7	Present the full search strategies for all databases, registers and websites, including any filters and limits used.	Supplemental text
Selection process	8	Specify the methods used to decide whether a study met the inclusion criteria of the review, including how many reviewers screened each record and each report retrieved, whether they worked independently, and if applicable, details of automation tools used in the process.	5
Data collection process	9	Specify the methods used to collect data from reports, including how many reviewers collected data from each report, whether they worked independently, any processes for obtaining or confirming data from study investigators, and if applicable, details of automation tools used in the process.	6
Data items	10a	List and define all outcomes for which data were sought. Specify whether all results that were compatible with each outcome domain in each study were sought (e.g. for all measures, time points, analyses), and if not, the methods used to decide which results to collect.	6
	10b	List and define all other variables for which data were sought (e.g. participant and intervention characteristics, funding sources). Describe any assumptions made about any missing or unclear information.	6
Study risk of bias assessment	11	Specify the methods used to assess risk of bias in the included studies, including details of the tool(s) used, how many reviewers assessed each study and whether they worked independently, and if applicable, details of automation tools used in the process.	6
Effect measures	12	Specify for each outcome the effect measure(s) (e.g. risk ratio, mean difference) used in the synthesis or presentation of results.	7
Synthesis methods	13a	Describe the processes used to decide which studies were eligible for each synthesis (e.g. tabulating the study intervention characteristics and comparing against the planned groups for each synthesis (item #5)).	6-7
	13b	Describe any methods required to prepare the data for presentation or synthesis, such as handling of missing summary statistics, or data conversions.	6-7
	13c	Describe any methods used to tabulate or visually display results of individual studies and syntheses.	7
	13d	Describe any methods used to synthesize results and provide a rationale for the choice(s). If meta-analysis was performed, describe the model(s), method(s) to identify the presence and extent of statistical heterogeneity, and software package(s) used.	7
	13e	Describe any methods used to explore possible causes of heterogeneity among study results (e.g. subgroup analysis, meta-regression).	7
	13f	Describe any sensitivity analyses conducted to assess robustness of the synthesized results.	-
Reporting bias assessment	14	Describe any methods used to assess risk of bias due to missing results in a synthesis (arising from reporting biases).	-
Certainty assessment	15	Describe any methods used to assess certainty (or confidence) in the body of evidence for an outcome.	-
**RESULTS**			
Study selection	16a	Describe the results of the search and selection process, from the number of records identified in the search to the number of studies included in the review, ideally using a flow diagram.	Page 8
	16b	Cite studies that might appear to meet the inclusion criteria, but which were excluded, and explain why they were excluded.	Supplemental Table S2
Study characteristics	17	Cite each included study and present its characteristics.	Supplemental Table S3

Section and Topic	Item #	Checklist item	Location where item is reported
Risk of bias in studies	18	Present assessments of risk of bias for each included study.	Page 10<br>Supplemental Table S5
Results of individual studies	19	For all outcomes, present, for each study: (a) summary statistics for each group (where appropriate) and (b) an effect estimate and its precision (e.g. confidence/credible interval), ideally using structured tables or plots.	Supplemental Table S2
Results of syntheses	20a	For each synthesis, briefly summarise the characteristics and risk of bias among contributing studies.	Page 10,<br>Supplemental figures 11-14
	20b	Present results of all statistical syntheses conducted. If meta-analysis was done, present for each the summary estimate and its precision (e.g. confidence/credible interval) and measures of statistical heterogeneity. If comparing groups, describe the direction of the effect.	Table 1, Figures 1-3,<br>Supplemental figures 2-10
	20c	Present results of all investigations of possible causes of heterogeneity among study results.	Page 10, Table 1<br>Figures 1-3,<br>Supplemental figures 2-10<br>Supplemental Table S4
	20d	Present results of all sensitivity analyses conducted to assess the robustness of the synthesized results.	-
Reporting biases	21	Present assessments of risk of bias due to missing results (arising from reporting biases) for each synthesis assessed.	-
Certainty of evidence	22	Present assessments of certainty (or confidence) in the body of evidence for each outcome assessed.	-
**DISCUSSION**			
Discussion	23a	Provide a general interpretation of the results in the context of other evidence.	Page 10-11
	23b	Discuss any limitations of the evidence included in the review.	Page 11
	23c	Discuss any limitations of the review processes used.	Page 11
	23d	Discuss implications of the results for practice, policy, and future research.	Page 12
**OTHER INFORMATION**			
Registration and protocol	24a	Provide registration information for the review, including register name and registration number, or state that the review was not registered.	Page 5
	24b	Indicate where the review protocol can be accessed, or state that a protocol was not prepared.	Page 5
	24c	Describe and explain any amendments to information provided at registration or in the protocol.	Page 5
Support	25	Describe sources of financial or non-financial support for the review, and the role of the funders or sponsors in the review.	Page 13
Competing interests	26	Declare any competing interests of review authors.	Page 13
Availability of data, code and other materials	27	Report which of the following are publicly available and where they can be found: template data collection forms; data extracted from included studies; data used for all analyses; analytic code; any other materials used in the review.	Page 13

Table S2. Studies excluded with their reason for exclusion.

Study	Title	Reason for exclusion
**U=U concept not clearly sought/ not clear definition**		
Agaku et al. 2022 [1]	A cross-sectional analysis of U=U as a potential educative Intervention to mitigate HIV stigma among youth living with HIV in South Africa	U=U concept sought with misleading definition
Siegel et al. 2019 [2]	Awareness and Perceived Effectiveness of HIV Treatment as Prevention Among Men Who Have Sex with Men in New York City	U=U not clearly sought
Patterson et al. 2017 [3]	Condomless Sex Among Virally Suppressed Women With HIV With Regular HIV-Serodiscordant Sexual Partners in the Era of Treatment as Prevention	U=U concept not clearly sought
Card et al. 2018 [4]	Belief in Treatment As Prevention and Its Relationship to HIV Status and Behavioral Risk	U=U concept not clearly sought
Holt et al. 2018 [5]	Comfort Relying on HIV Pre-exposure Prophylaxis and Treatment as Prevention for Condomless Sex: Results of an Online Survey of Australian Gay and Bisexual Men	U=U concept not clearly sought/ Only reported median score of belief in undetectable viral load effectiveness as a prevention method
Card et al. 2020 [6]	Substance use patterns and awareness of biomedical HIV prevention strategies among sexual and gender minority men in Canada	U=U concept not clearly sought
Coulibaly et al. 2023 [7]	Bridging the knowledge gap of biomedical HIV prevention tools among sub-saharan african immigrants in France. Results from an empowerment-based intervention	Not defined how knowledge about TasP was sought/ Also study on sub-Saharan African immigrants in general.
Couffignal et al. 2020 [8]	Treatment as prevention (TasP) and perceived sexual changes in behavior among HIV-positive persons: a French survey in infectious diseases departments in Paris	U=U not defined accurately
Coulibaly et al. 2023 [9]	Low knowledge of antiretroviral treatments for the prevention of HIV among precarious immigrants from sub-Saharan Africa living in the greater Paris area: Results from the Makasi project	U=U not defined accurately
Copeland et al. 2017 [10]	Disparities in HIV knowledge and attitudes toward biomedical interventions among the non-medical HIV workforce in the United States	U=U concept not sought
Lippman et al. 2020 [11]	Treatment as Prevention – Provider knowledge and counseling lag behind global campaigns	U=U not defined accurately
**No relevant data**		
Armstrong et al. 2018 [12]	Associations between Sexual Partner Number and HIV Risk Behaviors: Implications for HIV Prevention Efforts in a Treatment as Prevention (TasP) Environment	No relevant data
Brogan et al. 2019 [13]	Canadian results from the European Men-whohave-sex-with-men Internet survey (EMIS-2017)	No relevant data
Closson et al. 2019 [14]	HIV leadership programming attendance is associated with PrEP and PEP awareness among young, gay, bisexual, and other men who have sex with men in Vancouver, Canada	No relevant data
Tairy et al. 2018 [15]	Differences in knowledge, attitudes and behaviors of Israeli HIV-uninfected gaymen in HIV-discordant vs. concordant steady relationships	No relevant data
**No numerical data/ Overlapping data**		
Phanuphak et al. 2020 [16]	Implementing a Status-Neutral Approach to HIV in the Asia-Pacific	No numerical data provided
Rendina et al. 2018 [17]	Factors associated with perceived accuracy of the Undetectable = Untransmittable slogan among men who have sex with men: Implications for messaging scale-up and implementation	No numerical data provided
Cao et al 2021 [18]	Same-sex behavior disclosure to health care providers associated with greater awareness of pre-exposure prophylaxis	Overlap with included study
Bond et al. 2016 [19]	Good Health and Moral Responsibility: Key Concepts Underlying the Interpretation of Treatment as Prevention in South Africa and Zambia Before Rolling Out Universal HIV Testing and Treatment	No numerical data provided
Grace et al. 2023 [20]	Gay, bisexual, and queer men's confidence in the Undetectable equals Untransmittable HIV prevention message: longitudinal qualitative analysis of the sexual decision-making of pre-exposure prophylaxis users over time	No numerical data provided - Qualitative study
**Studies conducted before U=U was implemented**		
Bavinton et al 2016 [21]	Willingness to Act upon Beliefs about 'Treatment as Prevention' among Australian Gay and Bisexual Men	Study was conducted before U=U was disseminated.
Sharma et al 2018 [22]	Birth Cohort Variations Across Functional Knowledge of HIV Prevention Strategies, Perceived Risk, and HIV-Associated Behaviors Among Gay, Bisexual, and Other Men Who Have Sex With Men in the United States	Study was conducted before U=U was disseminated.
El-Sadr et al 2016 [23]	Prevention for HIV-infected Persons in HPTN 065:Room for Improvement	Study was conducted before U=U was disseminated
Prati et al. 2016 [24]	PEP and TasP Awareness among Italian MSM, PLWHA, and High-Risk Heterosexuals and Demographic, Behavioral, and Social Correlates	Study was conducted before U=U was disseminated.
Carter et al. 2015 [25]	Gay and bisexual men's awareness and knowledge of treatment as prevention: findings from the Momentum Health Study in Vancouver, Canada	Study was conducted before U=U was disseminated.

Holt et al. 2014 [26]	Australian Gay and Bisexual Men's Attitudes to HIV Treatment as Prevention in Repeated, National Surveys, 2011-2013	Study was conducted before U=U was disseminated.
Holt et al. 2016 [27]	Increasing Belief in the Effectiveness of HIV Treatment as Prevention: Results of Repeated, National Surveys of Australian Gay and Bisexual Men, 2013–15	Study was conducted before U=U was disseminated.

Table S3. U=U awareness and acceptance data in eligible studies

First author (year)	Participant Number	Region	Mean age (Age range)	Population	Main Outcome Reported	Percentage
**MSM**						
Agarwal et al 2023[28]	3126	India	28 years (Median age)	MSM, TW, TM and other adults that have sex with men (excluding cisgender women and those born as women) (3.1%, HIV +)	**U=U awareness** ("a little familiar" "somewhat familiar" and "I am familiar" answers coupled, calculated by subtracting "not at all familiar" answers from the total)<br>**U=U perceived** as:<br>-completely accurate<br>-completely or partially accurate after explanation was provided	1206/3126 (39%)<br><br><br>782/3126 (25%)<br>1141/3126 (36.5%)
Avelino Silva et al 2022[29]	55924	Latin America	28 years (Median age) (range 18–81)	MSM (99%) and trans men (16% HIV+)	**U=U knowledge** ("I knew this already" for the U=U statement)	28917/55924 (52%)
Ayala et al 2018[30]	1118 (HIV positive subset)	USA based analysis with global data	Majority was <29	"Hornet" application users (the U=U question was asked only in HIV positive participants) (All PLWH)	**U=U awareness** (Are you aware that if you are undetectable, it's virtually impossible to transmit HIV?)	904/1118 (80.9%)
Cadelina et al 2019[31]	415	Philippines	NR	MSM (No info on HIV status)	**U=U awareness** (knowledge on the new concept of undetectable equals untransmittable, where in a person living with HIV who have undetectable viral load by taking antiretroviral drugs cannot transmit HIV sexually)	104/415 (25%)
Cao et al 2021[32]	689	China	30 years (Mean age) (SD:11)	MSM negative or unknown HIV status (No HIV + participants)	**U=U knowledge** (Proportion who answered "correct" in the U=U statement)	138/689 (20%)
Card et al 2021 [33]*know	2681	Canada	35.29 (Mean age) (SD:12.76)	Sexual Gay minority men (7.1% HIV+)	**U=U knowledge** (Proportion who answered "Yes I knew this already" in the U=U statement)	1946/2681 (72.6%)
Carneiro et al 2021[34]	3286	USA	Majority was under 30 years old	HIV negative MSM, TW and TM who have sex with men) (No HIV + participants)	**U=U knowledge** ("Yes" or "No" in the question "Have you heard of the statement Undetectable = Untransmittable or U=U"<br>**Trust in the U=U message** among those who were aware of it (Answered as "Very trustworthy" or "Trustworthy" in the U=U message)	2809/3286 (85.5%)<br><br>1187/2809 (42.3%)
Chinbunchorn et al 2023[35]	960	Thailand	34 years (Median age)	Users of "Hornet" application (80.4% MSM) (17.7% HIV+)	**U=U perceived** as:<br>-completely accurate<br>-completely or somewhat accurate	 69/302 (22.8%)<br>159/302 (52.6%)
Clement et al 2019[36] *strongly agree	678	USA	43 years (Average age)	MSM (15% HIV+)	**U=U perception** as the proportion of participants that:<br>- "Agreed a lot" with U=U slogan<br>- "Agreed a lot" or "agreed a litte"	 161/660 (24%)<br>315/660 (47.7%)
Coyne et al 2022b[37]	66	Ireland	22.6 (Mean age) (Sd: 6.38)	Subset of MSM that finished the survey (No info on HIV status)	**U=U awareness** (Not further defined)	56/66 (85%)
Ferreira et al 2022a[38]	2552	Brazil	35.1 years (Mean age)	Majority were MSM (98%) (31% HIV+)	**U=U perception** as:<br>- "Completely accurate"<br>- "Completely accurate" or "accurate"	 1600/2552 (62.7%)<br>2055/2552 (80.5%)
MacGibbon et al 2023[39]*	1280	Australia	38 (Median age) (IQR: 30-52)	MSM, queer and non-binary people (7.4% HIV+)	**U=U awareness** ("a little familiar", "somewhat familiar" and "very familiar" answers coupled)	1006/1280 (78.6%)

First author (year)	Participant Number	Region	Mean age (Age range)	Population	Main Outcome Reported	Percentage
					**U=U perceived** as:<br>- "completely accurate"<br>- "completely accurate" or "accurate"	 284/1006 (28.2%)<br>677/1006 (67.3%)
McKay et al 2022[40]* Reported both overall MSM and MSM PLWH	633	USA	59.4 (sd: 6.3)	Older MSM (21% HIV+)	**U=U awareness** ("Yes" or "No" answer in the "Have you heard about the U=U question)<br>**U=U believing** (Following a short description of the U=U the percentage of participants that ranked it as:<br>- "very believable"<br>- "very believable" or "somewhat believable"	160/633 (25.3%)<br><br>222/633 (35.1%)<br>391/633 (61.8%)
Meanley et al 2019[41]	264	USA	22.44 (Mean Age) (sd: 2.02)	Young MSM (negative or unknown)	**U=U awareness** ("a little familiar", "somewhat familiar" and "very familiar" answers coupled)<br>**U=U perceived accuracy:**<br>- "completely accurate"<br>- "completely accurate" or "somewhat accurate"	184/264 (69.7%)<br><br>70/184 (38%)<br>149/184 (81%)
Meunier et al 2020[42]	170	USA	28.5 (Median Age)	MSM who had engaged in exchange sex (negative or unknown)	**U=U perception** (Combining stages 3,4 and 5 that included participants that trusted and agreed with TasP is completely effective at preventing HIV transmission)	122/170 (71.8%)
Rendina et al 2020a[43]	17147	USA	40.4 years (mean age)	Adolescent and adult Sexual Minority Men PLWH	**U=U awareness** (The proportion that had heard of U=U slogan)	12107/17147 (70.6%)
Rendina et al 2020b[44]*	111747	USA	NR 99% MSM	The majority (99%) were MSM (14.7% HIV+)	**U=U perception** (Percentage of those who perceived U=U as<br>- "completely accurate"<br>- "completely accurate" or "somewhat accurate"	 24654/111747 (22.1%)<br>61596/111747 (55.1%)
Reyes-Diaz et al 2020[45]	10265	18 Latin American Countries	NR MSM	MSM living with HIV	**U=U awareness** ("knew about U=U")	7566/10265 (74%)
Torres et al 2020 [46]	785 (MSM subset)	Brazil	Majority >35 years (56.8%) MSM	MSM (HIV negative/ unknown)	**U=U perception** perceived as<br>- "completely accurate"<br>- "completely accurate" or "partially accurate" (calculated approximately from figure)	 347/785 (44.2%)<br>488/785 (62%)
Wilkinson et al 2018 [47]	462	Australia	32 (Median age) (IQR: 25-41) 99% MSM	HIV-Negative MSM not using PrEP (HIV-negative)	**U=U perception.** In response to the statement "A person with an undetectable viral load cannot pass on HIV" the proportion that:<br>- "strongly agreed"<br>- "strongly agreed" or "agreed"	 <br>13/462 (3%)<br>83/462 (18%)
Zhang et al 2023[48]	490	China	NR	HIV infected MSM (all MSM living with HIV)	**U=U awareness** (were aware of U=U)<br>**U=U perception** (accepted U=U)	295/490 (60.2%)<br>248/490 (50.6%)
**PLWH**						
Adams et al 2019[49]	81	UK	NR	PLWH on ART and undetectable (no info on sexual orientation)	**U=U awareness** (Heard U=U)<br>**U=U perception** (agreement with "being undetectable for six months means no sexual transmission")	55/81 (68%)<br>65/81 (80%)
Ayala et al 2018[30]	1118 (HIV positive subset)	USA based analysis with global data	Majority was <29	"Hornet" application users (the U=U question was asked only in HIV positive participants)	**U=U awareness** (Are you aware that if you are undetectable, it's virtually impossible to transmit HIV?)	904/1118 (80.9%)

First author (year)	Participant Number	Region	Mean age (Age range)	Population	Main Outcome Reported	Percentage
				(participants from gay social app)		
Card et al 2021 [33]*know	2681	Canada	35.29 (Mean age) (SD:12.76)	Sexual Gender minority men	**U=U knowledge** (Proportion who answered "Yes I knew this already" in the U=U statement)	182/190 (96%)
Cingolani et al 2023 [50]	397(subset of People living with HIV)	Italy	Majority (61.9%) was <50 years	Web survey participants (33% Heterosexual)	**U=U awareness** ("Have you heard of U=U?")<br>**U=U perception**<br>- "completely accurate"<br>- "completely accurate" or "enough accurate"	296/397 (74.6%)<br><br>238/296 (80%)<br>292/296 (99%)
Clement et al 2019[36] *strongly agree	678	USA	43 years (Average age)	HIV+ subset of MSM	**U=U perception** as the proportion of participants that:<br>- "Agreed a lot" with U=U slogan	 45/102 (44%)
Forbes et al 2018[51]	86	UK	47 (Median age) (22-71)	Heterosexual PLWH	**U=U awareness** ("Have you heard about U=U?")	34/86 (40%)
Garcia et al 2023[52]	169	Spain	46.6 (mean age) (sd:12.2)	Adult PLWH (no info on sexual orientation)	**U=U awareness** (Participants that answered "TRUE" to the following statement "There is no risk of transmission when the viral load is undetectable")	109/169 (64.5%)
Huntingdon et al 2020[53]	139	Australia	45	Adult PLWH (24.5% Heterosexual)	**U=U perception** (Proportion of the participants that in response to that statement ''a person with undetectable viral load cannot pass on HIV" stated that they:<br>- "strongly agreed"<br>- "strongly agreed" or "agreed"	 <br><br>20/45 (44.6%)<br>32/45 (70.5%)
MacGibbon et al 2023[39]*	1280	Australia	38 (Median age) (IQR: 30-52)	MSM, queer and non-binary people (95, 7.4% Heterosexual)	**U=U awareness** ("a little familiar", "somewhat familiar" and "very familiar" answers coupled)	91/95 (96%)
McKay et al 2022[40]*	633	USA	59.4 (sd: 6.3)	Older MSM	**U=U awareness** ("Yes" or "No" answer in the "Have you heard about the U=U question)<br>**U=U perception** (following a short description of the U=U the percentage of participants that ranked it as:<br>- "Very believable"<br>- "Very believable" or "Somewhat believable"	73/131 (55.7%)<br><br><br><br>78/135 (57.7%)<br>102/135 (75.5%)
Okoli et al 2021[54]	2389	25 countries (mostly USA and Europe)	Majority (70.7%) was <50 years	PLWH (40.2% Heterosexual)	**U=U awareness** (calculated as the total minus those that were completely unaware)	2093/2389 (87.6%)
Rendina et al 2020a[43]	17147	USA	40.4 years (mean age)	Adolescent and adult PLWH (0.3% Heterosexual)	**U=U awareness** (The proportion that had heard of U=U slogan)	12107/17147 (70.6%)
Rendina et al 2020b[44]	16392	USA	NR	HIV positive subset of Sexual Minority Men sample (0.8% Heterosexual)	**U=U perception** as:<br>- "completely accurate"<br>- "completely accurate" or "somewhat accurate"	 8403/16392 (51.3%)<br>13740/16392 (83.8%)
Reyes-Diaz et al 2020[45]	10265	18 Latin American Countries	NR	HIV+ MSM	**U=U awareness** ("knew about U=U")	7566/10265 (74%)
Stutterheim et al 2022[55]	194(subset of the 2020 sample)	The Netherlands	49.4 (mean age) (sd: 12)	PLWH (29.5% Heterosexual)	**U=U awareness** ("had heard of U=U")	190/194 (97.9%)

First author (year)	Participant Number	Region	Mean age (Age range)	Population	Main Outcome Reported	Percentage
Torres et al 2020[46]	347 (subset of PLWH)	Brazil	Majority (56.8%) was >35 years	PLWH (557, 33.1% Heterosexual)	**U=U perception** as<br>- "completely accurate"<br>- "completely accurate" or "partially accurate"	 274/347 (79%)<br>312/347 (90%)
Zhang et al 2023[48]	490	China	490	HIV infected MSM	**U=U awareness** (were aware of U=U)<br>**U=U perception** (accepted U=U)	295/490 (60.2%)<br>248/490 (50.6%)
**Healthcare Professionals**						
Cingolani et al 2023[50]	90	Italy	Majority (54.4%) was <40	Infectious diseases physicians	**U=U awareness** ("Have you heard of U=U?")<br>**U=U perception** as:<br>- "completely accurate"<br>- "completely accurate" or "enough accurate"	83/90 (92%)<br><br>66/83 (79%)<br>79/83 (95%)
Clifford et al 2021[56]	22	UK	NR	22 respondents: 17 female, 5 male; 8 dentists, 6 hygienists, 3 nurses)	**U=U awareness** (Percentage that recognized that an undetectable viral load rendered HIV untransmittable sexually.)	6/22 (27%)
Keane et al 2020[57]	139	UK	NR	Junior doctors	**U=U awareness** (Proportion that had heard of U=U)	92/139 (66%)
Mastrogianni et al. 2021[58]	339	Greece	39.4 (sd: 9.7)	Hospital Care Workers (60.8% were doctors)	**U=U awareness** (Proportion that knew that PLHIV receiving HAART with an undetectable viral load do not transmit the virus)	185/339 (54.6%)
Nunes et al 2023 [59]	197	Brazil	NR	Physicians	**U=U awareness** (Have you heard of U=U?)<br>**U=U perception** as those who:<br>- strongly agreed with U=U<br>- strongly agreed or agreed with U=U	102/195 (52%)<br><br>74/195 (38%)<br>144/195 (73%)
Ripamonti et al 2022[60]	286	Italy	46 (Median age) (IQR: 34-58)	Infectious disease clinicians	**U=U perception** (The proportion that deems zero risk of HIV transmission through condomless sex with undetectable patients)	144/286 (50.3%)
Shongwe et al 2021[61]	271	Egypt	29 years (mean age)	Healthcare professionals (18% Medical Specialized, 18% Surgical, 9% infectious diseases specialists, 38% non-specialized)	**U=U awareness** (The total minus the ones that had not heard of U=U)<br>**U=U perception** (The total minus those who thought that a PLWH with an undetectable viral load can still pass the virus to a negative sexual partner)	60/271 (22%)<br><br>125/271 (46%)
Wu et al 2023[62]	407	Australia	39.7 (mean age) (sd: 8.4)	General Practitioners	**U=U perception** as the proportion that:<br>- "strongly agreed" with U=U<br>- "strongly agreed" or "agreed" with U=U	 137/407 (33.7%)<br>302/407 (74.2%)
**General Population**						
Colpani et al 2021[63]	411	Italy	NR	General Population of Italy	**U=U awareness** ("Knew the U=U campaign)<br>**U=U perception** (The ones that did not believe that people with HIV with undetectable HIV-RNA could transmit the virus)	60/411 (14.6%)<br>161/411 (39.1%)
Coyne et al 2022a[64]	707	UK	44.36 (Mean age) (Sd: 15.38)	Sample Representative of the general population	**U=U awareness** (The proportion that answered "I knew this already" after being provided with the U=U definition)	82/707 (11.6%)
Coyne et al 2022b[37]	314	Ireland	22.6 (Mean age) (Sd: 6.38)	Participants that finished an online survey (60.55% female)	**U=U awareness** (Not further defined)	156/314 (49.7%)

First author (year)	Participant Number	Region	Mean age (Age range)	Population	Main Outcome Reported	Percentage
De Vito et al 2022[65]	2183	Italy	39.8 (Mean age) (sd:12.8)	General Population	**U=U awareness** (knowledge of the U=U campaign)	356/2183 (16.3%)
Ferreira et al 2022b[66]	401	Brazil	Majority (52.4%) were <39 years	Adult Brazilians	**U=U perception** as<br>- "completely accurate"<br>- "completely accurate" or "accurate"	 128/401 (31.9%)<br>197/401 (49.1%)
Katsarolis et al 2021[67]	1954	Greece	NR (Participants aged > 18 years)	Random sample of the general population	**U=U awareness** (Participants who answered "true" to the following "PLWH with an undetectable viral load do not transmit the virus to their partners")	391/1954 (20%)
Htun et al 2023[68]	582	UK	39 (Median)	People attending a hospital in the world AIDS day	**U=U awareness** (100% minus the proportion that was not aware of the U=U message<br>**U=U perception** (100% - the proportion of participants that thought that HIV can transmit to the partner even on treatment)	58/582 (10%)<br><br>291/582 (50%)
Miscellaneous						
Cingolani et al 2023[50]	634	Italy	Majority (72.7%) was <40	HIV negative people having unprotected sex (68% male)	**U=U awareness** ("Have you heard of U=U?")<br>**U=U perception** as:<br>- "completely accurate"<br>- "completely accurate" or "enough accurate"	297/634 (46.8%)<br><br>200/297 (67.3%)<br>290/297 (97.6%)
Rivera et al 2021[69]	485	USA	Majority (41%) was 18-29	Heterosexually active black and latino adults	**U=U awareness** (Participants who answered "Yes" to the following "A person with HIV whose virus is undetectable cannot pass HIV to their sex partners. Have you heard this before?")	170/485 (35%)
Smith et al 2021[70]	180 (68 in the standard of care (SOC) days and 112 in the U=U days)	South Africa	35 (median) (IQR: 27-45)	Men presenting for an HIV test	**U=U awareness** (prior knowledge)	126/180 (70%) (coupled)<br>41/68 (60%) (SOC days)<br>85/112 (76%) (U=U days)
Torres et al 2020[46]	558	Brazil	Majority (56.8%) was >35 years	HIV negative/ unknown other populations (Majority: heterosexual cisgender women)	**U=U perception** as:<br>- "completely accurate"<br>- "completely accurate" and "partially accurate"	 96/558 (17.2%)<br>173/558 (31%)

AIDS: acquired immunodeficiency syndrome; HIV: Human immunodeficiency virus; IQR: Interquartile range; MSM: Men who have sex with men; NR: Not reported PLWH: People living with HIV; sd: standard deviation; TM: Transgender Men; TW: Transgender Women UK: United Kingdom; USA: United states of Amercia

Table S4. Meta-regression analysis examining the role of publication year and PLWH percentage as a potential modifier on U=U awareness, complete acceptance and any acceptance in MSM and the role of publication year and sexual orientation as a potential modifier on U=U awareness in PLWH.

Outcomes	n§	Coefficient (95%CI)	P
**Outcome: U=U Awareness in MSM**			
Publication Year (per 1 year)	14	-0.05 (-0.23 – 0.13)	0.553
PLWH percentage (per 1 %)	12	0.003 (-0.004 – 0.01)	0.33
**Outcome: U=U complete acceptance MSM**			
Publication Year (per 1 year)	11	0.05 (-0.131 – 0.23)	0.552
PLWH percentage (per 1 %)	11	0.008 (-0.024 – 0.039)	0.595
**Outcome: U=U any acceptance MSM**			
Publication Year (per 1 year)	12	0.044 (-0.1 – 0.19)	0.513
PLWH percentage (per 1 %)	12	0.0012 (-0.008 – 0.011)	0.787
**Outcome: U=U Awareness in PLWH**			
Publication Year (per 1 year)	13	0.73 (-0.73 – 0.22)	0.295
Sexual orientation (per 1 %)	11	-0.005 (-0.016 – 0.005)	0.280

CI: confidence interval; MSM: Men who have sex with men; PLWH: People living with HIV

Table S5. Evaluation of the eligible studies with Newcastle-Ottawa scale adapted for cross-sectional studies.

Study	Selection				Comparability	Outcome		Total
	**Representativeness of the cases**	**Sample Size (Satisfactory > 200)**	**Non-response rate**	**Ascertainment of the screening/surveillance tool:**	**Comparability on age and other factors**	**Assessment of outcome**	**Statistical test**	
Adams (2019)[49]	1	0	0	1	0	1	0	3
	PLWH on ART and undetectable	81	No info	Full description of a non-validated tool	Potential confounders were not sought	Self-Report	The data has not undergone any analysis.	
Agarwal (2023)[28]	1	1	1	1	0	1	0	5
	Individuals Having sex with Men using a social App	3126	Response Rate 100%	Full description of a non-validated tool	Potential confounders were sought but we included unadjusted data.	Self-Report	Full description of statistical test but we included unadjusted data	
Avelino-Silva (2022)[29]	1	1	1	1	0	1	0	5
	MSM from a multicenter internet-based survey	55924	Response Rate 100%	Full description of a non-validated tool	Potential confounders were sought but we included unadjusted data.	Self-Report	Full description of statistical test but we included unadjusted data	
Ayala (2018)[30]	1	1	1	1	0	1	0	5
	Users of a social App	1118	Response Rate 100%	Full description of a non-validated tool	Potential confounders were sought but we included unadjusted data.	Self-Report	Full description of statistical test but we included unadjusted data	
Cadelina (2019)[31]	1	1	0	1	0	1	0	4
	Non-probability sampling of MSM.	415	Not reported	Full description of a non-validated tool	Potential confounders were not sought for the included outcomes	Self-Report	The data has not undergone any analysis.	
Cao (2021)[32]	1	1	1	1	0	1	0	5
	MSM recruited through an NGO	689	Response Rate 100%	Full description of a non-validated tool	Potential confounders were sought but we included unadjusted data.	Self-Report	Full description of statistical test but we included unadjusted data	
Card (2021)[33]	1	1	1	1	0	1	0	5
	SGMM recruited from Pride festivals	2681	Response Rate 100%	Full description of a non-validated tool	Potential confounders were sought but we included unadjusted data.	Self-Report	Full description of statistical test but we included unadjusted data	
Carneiro (2021)[34]	1	1	0	1	0	1	0	4
	MSM and TW from an internet-based cohort	3286	Not Reported	Full description of a non-validated tool	Potential confounders were sought but we included unadjusted data.	Self-Report	Full description of statistical test but we included unadjusted data	
Chinbunchorn (2023)[35]	1	1	0	1	0	1	0	4
	Users of a social App	590	Response Rate varied (50% and 86%)	Full description of a non-validated tool	Potential confounders were sought but we included unadjusted data.	Self-Report	Full description of statistical test but we included unadjusted data	
Cingolani (2023)[50]	1	1	1	1	0	1	0	5
	Nationwide survey in centers and online	1112	Response Rate 100%	Full description of a non-validated tool	Potential confounders were sought but we included unadjusted data.	Self-Report	Full description of statistical test but we included unadjusted data	
Clement (2019)[36]	1	1	0	1	0	1	0	4
	MSM using a dating app	969	Not Reported	Full description of a non-validated tool	Potential confounders were sought but we included unadjusted data.	Self-Report	Description of statistical test but we included unadjusted data	

Study	Selection				Comparability	Outcome		Total
	**Representativeness of the cases**	**Sample Size (Satisfactory > 200)**	**Non-response rate**	**Ascertainment of the screening/surveillance tool:**	**Comparability on age and other factors**	**Assessment of outcome**	**Statistical test**	
Clifford (2021)[56]	0	0	1	1	0	1	0	3
	Workers in dental settings (not representative of healthcare professionals)	22	Response Rate 100%	Full description of a validated tool	Potential confounders were not sought for the included outcomes	Self-Report	The data has not undergone any analysis.	
Colpani (2021)[63]	1	1	1	0	0	1	0	4
	General Italian Population	413	Response Rate 99%	Insufficient description of measurement tool	Potential confounders were not sought for the included outcomes	Self-Report	The data has not undergone any analysis	
Coyne (2022)a[64]	1	1	1	1	0	1	0	5
	Nationally representative UK sample	707	Response Rate 100%	Full description of a non-validated tool	No adjustment for confounders for the included outcomes	Self-Report	The data has not undergone any analysis	
Coyne (2022)b[37]	1	1	1	1	0	1	0	5
	Online Survey participants (General Population)	314	Response Rate 100%	Full description of a non-validated tool	Potential confounders were not sought for the included outcomes	Self-Report	The data has not undergone any analysis	
De Vito (2022)[65]	1	1	0	1	0	1	0	4
	General Population of Italy (Web-based Survey)	2183	Not Reported	Full description of a non-validated tool	Potential confounders were not sought for the included outcomes	Self-Report	The data has not undergone any analysis	
Ferreira (2022)a[38]	1	1	1	1	0	1	0	5
	Sexual gender minority men through social apps	2552	Response Rate 100%	Full description of a non-validated tool	Potential confounders were not sought for the included outcomes	Self-Report	Full description of statistical test but we included unadjusted data	
Ferreira (2022)b[66]	1	1	1	1	0	1	0	5
	MSM recruited through an online survey	401	Response Rate 100%	Full description of a non-validated tool	Potential confounders were not sought for the included outcomes	Self-Report	Description of statistical test but we included unadjusted data	
Forbes (2018)[51]	1	0	0	1	0	1	0	3
	PLWH recruited by a support organization	86	Not Reported	Full description of a non-validated tool	Potential confounders were not sought	Self-Report	The data has not undergone any analysis	
Garcia (2023)[52]	1	0	0	1	0	1	0	3
	PLWH recruited by 5 centers	169	Not Reported	Full description of a non-validated tool	Potential confounders were not sought for the included outcomes	Self-Report	The relevant data has not undergone any analysis	
Htun (2023)[68]	1	1	0	1	0	1	0	4
	People attending a hospital in World AIDS day.	582	Not Reported	Insufficient description of measurement tool	Potential confounders were not sought	Self-Report	The data has not undergone any analysis	
Huntingdon (2020)[53]	1	0	0	1	0	1	0	3
	PLWH recruited through clinics or community	139	Not Reported	Full description of a non-validated tool	Potential confounders were sought but we included unadjusted data.	Self-Report	Description of statistical test but we included unadjusted data	
Katsarolis (2021)[67]	1	1	0	1	0	1	0	4
	Random sampling of the general population	1954	Not Reported	Full description of a non-validated tool	Potential confounders were not sought	Self-Report	The data has not undergone any analysis	

Study	Selection				Comparability	Outcome		Total
	**Representativeness of the cases**	**Sample Size (Satisfactory > 200)**	**Non-response rate**	**Ascertainment of the screening/surveillance tool:**	**Comparability on age and other factors**	**Assessment of outcome**	**Statistical test**	
Keane (2020)[57]	1	0	1	1	0	1	0	4
	Web-Based survey for junior doctors	139	Response Rate 96%	Description of a non-validated tool	Potential confounders were not sought	Self-Report	The data has not undergone any analysis	
MacGibbon (2023)[39]	1	1	1	1	0	1	0	5
	MSM from web-based survey	1280	Response Rate 100%	Full description of a non-validated tool	Potential confounders were sought but we included unadjusted data.	Self-Report	Description of statistical test but we included unadjusted data	
Mastrogianni (2021)[58]	1	1	0	1	0	1	0	4
	Healthcare workers from 3 different hospitals	339	Not Reported	Description of a non-validated tool	Potential confounders were not sought	Self-Report	The data has not undergone any analysis	
McKay (2022)[40]	1	1	0	1	0	1	0	4
	Older adults recruited either online or in venues	1256	Not Reported	Full description of a non-validated tool	Potential confounders were sought but we included unadjusted data.	Self-Report	Description of statistical test but we included unadjusted data	
Meanley (2019)[41]	1	1	0	1	0	1	0	4
	MSM recruited online	264	Not Reported	Full description of a non-validated tool	Potential confounders were sought but we included unadjusted data.	Self-Report	Description of statistical test but we included unadjusted data	
Meunier (2020)[42]	1	0	1	1	0	1	0	4
	Online survey on MSM engaging in exchange sex	170	Response Rate 100%	Full description of a non-validated tool	Potential confounders were not sought	Self-Report	The data has not undergone any analysis	
Neves (2023)[59]	1	0	1	1	0	1	0	4
	Doctors working in a hospital	197	Response Rate ≈ 100%	Full description of a non-validated tool	Potential confounders were sought but we included unadjusted data.	Self-Report	Description of statistical test but we included unadjusted data	
Okoli (2021)[54]	1	1	1	1	0	1	0	5
	Web based survey for PLWH in 25 countries	2389	Response Rate 100%	Description of a non-validated tool	Potential confounders were not sought for the relevant outcomes	Self-Report	Description of statistical test but we included unadjusted data	
Rendina (2020)a[43]	1	1	1	1	0	1	0	5
	Web-based survey on men living with HIV	30361	Response Rate 100%	Description of a non-validated tool	Potential confounders were not sought for the relevant outcomes	Self-Report	Description of statistical test but we included unadjusted data	
Rendina (2020)b[44]	1	1	1	1	0	1	0	5
	Web-based survey on sexual minority men	111747	Response Rate 100%	Full description of a non-validated tool	Potential confounders were sought but we included unadjusted data.	Self-Report	Description of statistical test but we included unadjusted data	
Reyes-Diaz (2020)[45]	1	1	0	1	0	1	0	4
	Web-based survey for MSM	10265	Not Reported	Full description of a non-validated tool	Potential confounders were sought but we included unadjusted data.	Self-Report	Description of statistical test but we included unadjusted data	
Ripamonti (2022)[60]	1	1	1	1	0	1	0	5
	Online survey on clinicians working in a group of hospitals	286	PrEP was provided to all the participants	Full description of a non-validated tool	Potential confounders were not sought for the relevant data	Self-Report	The relevant data has not undergone any analysis	
Rivera (2021)[69]	1	1	1	1	0	1	0	5

Study	Selection				Comparability	Outcome		Total
	**Representativeness of the cases**	**Sample Size (Satisfactory > 200)**	**Non-response rate**	**Ascertainment of the screening/surveillance tool:**	**Comparability on age and other factors**	**Assessment of outcome**	**Statistical test**	
	Heterosexually active adults recruited through respondent-driven sampling	485	Response Rate 100%	Full description of a non-validated tool	Potential confounders were sought but we included unadjusted data.	Self-Report	Description of statistical test but we included unadjusted data	
Shongwe (2021)[61]	1	1	0	1	0	1	0	5
	Web-based survey for healthcare professionals in Cairo university hospitals	271	Not reported	Full description of a non-validated tool	Potential confounders were not sought for the relevant data	Self-Report	The relevant data has not undergone any analysis	
Smith (2021)[70]	1	0	1	1	0	1	0	4
	Men presenting for an STI test	180	Response Rate 100%	Full description of a non-validated tool	Potential confounders were sought but we included unadjusted data.	Self-Report	Description of statistical test but we included unadjusted data	
Stutterheim (2022)[55]	1	0	1	1	0	1	0	4
	Online survey for PLWH	194	Response Rate 100%	Description of a non-validated tool	Potential confounders were not sought for the relevant data	Self-Report	The relevant data has not undergone any analysis	
Torres (2020)[46]	1	1	1	1	0	1	0	5
	Participants recruited in web-based survey from social platforms	1690	Response Rate 100%	Description of a non-validated tool	Potential confounders were sought but we included unadjusted data.	Self-Report	Description of statistical test but we included unadjusted data	
Wiklinson (2018)[47]	1	1	1	1	0	1	0	5
	Online survey on adult GBM not using PrEP	462	Response Rate 100%	Description of a non-validated tool	Potential confounders were not sought for the relevant data	Self-Report	The relevant data has not undergone any analysis	
Wu (2023)[62]	1	1	1	1	0	1	0	5
	Online survey for GPs working in Australia	407	Response Rate 100%	Description of a non-validated tool	Potential confounders were sought but we included unadjusted data.	Self-Report	Description of statistical test but we included unadjusted data	
Zhang (2023)[48]	1	1	1	1	0	1	0	5
	MSM receiving ART using a convenience sampling method	490	Response Rate 100%	Insufficient description of measurement tool	Potential confounders were sought but we included unadjusted data.	Self-Report	Description of statistical test but we included unadjusted data	

Supplemental Figures

Figure S1. PRISMA 2020 flow chart presenting the successive steps in the selection of eligible studies.

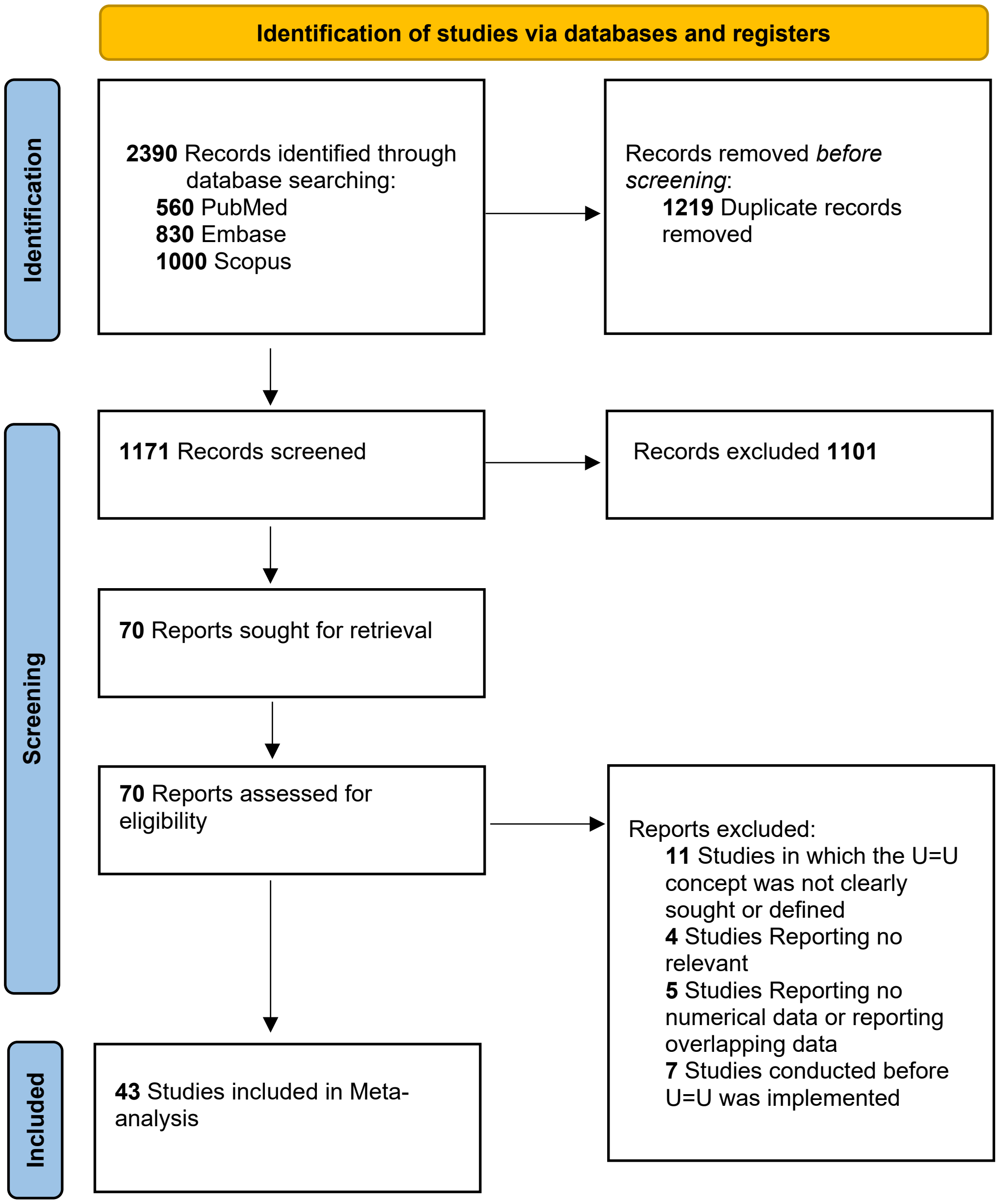

Figure S2: Forest plot describing the prevalence of U=U awareness in MSM. Subgroup analyses by the percentage of PLWH is presented

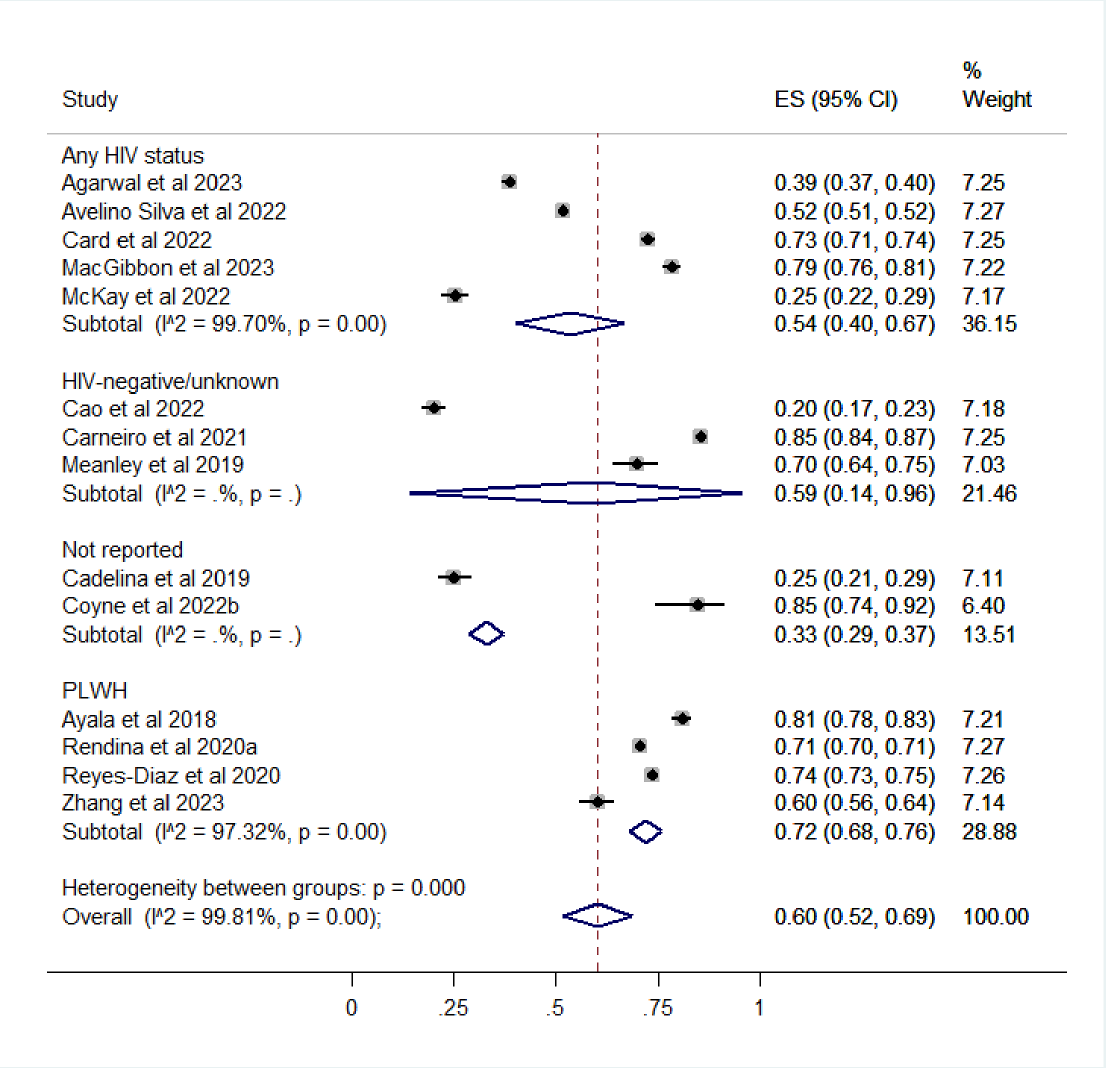

Figure S3: Forest plot describing the prevalence of complete U=U acceptance in MSM. Subgroup analyses by the percentage of PLWH is presented

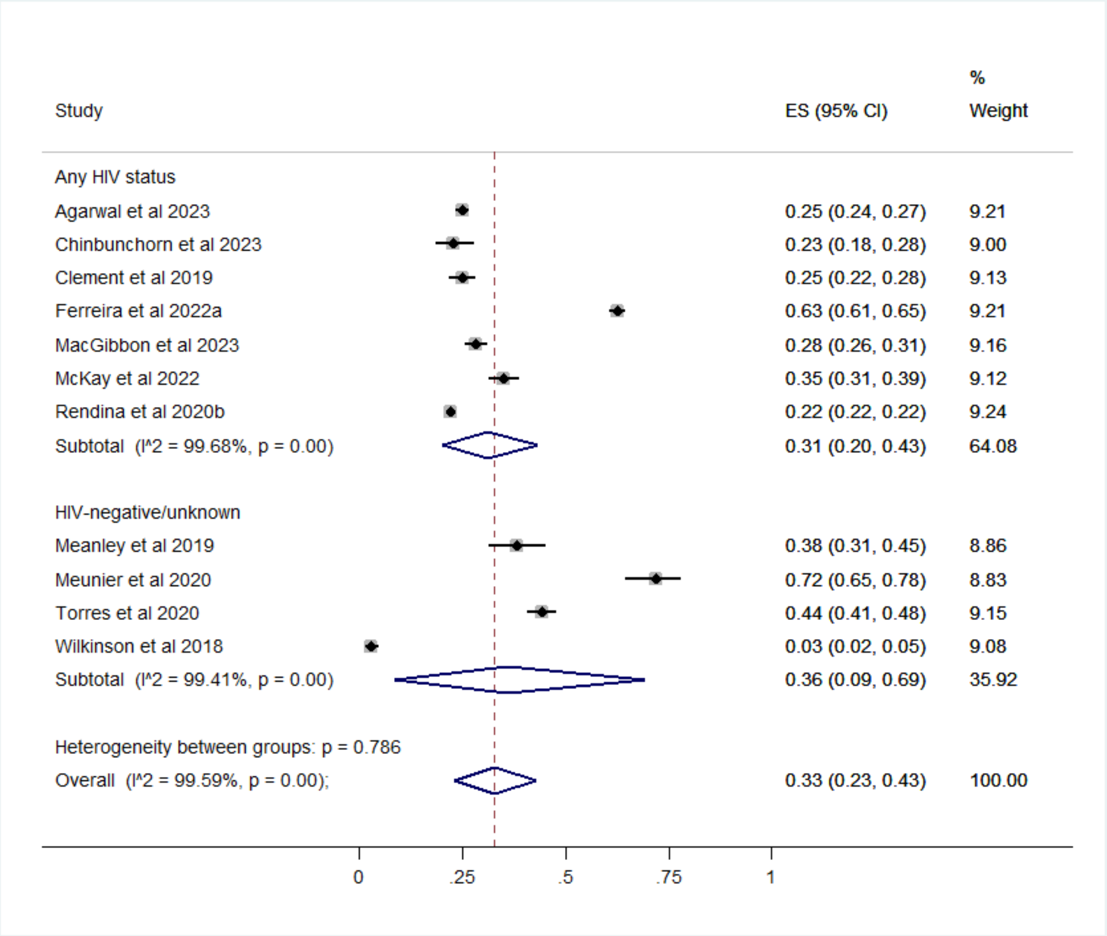

Figure S4: Forest plot describing the prevalence of any U=U acceptance in MSM. Subgroup analyses by the percentage of PLWH is presented

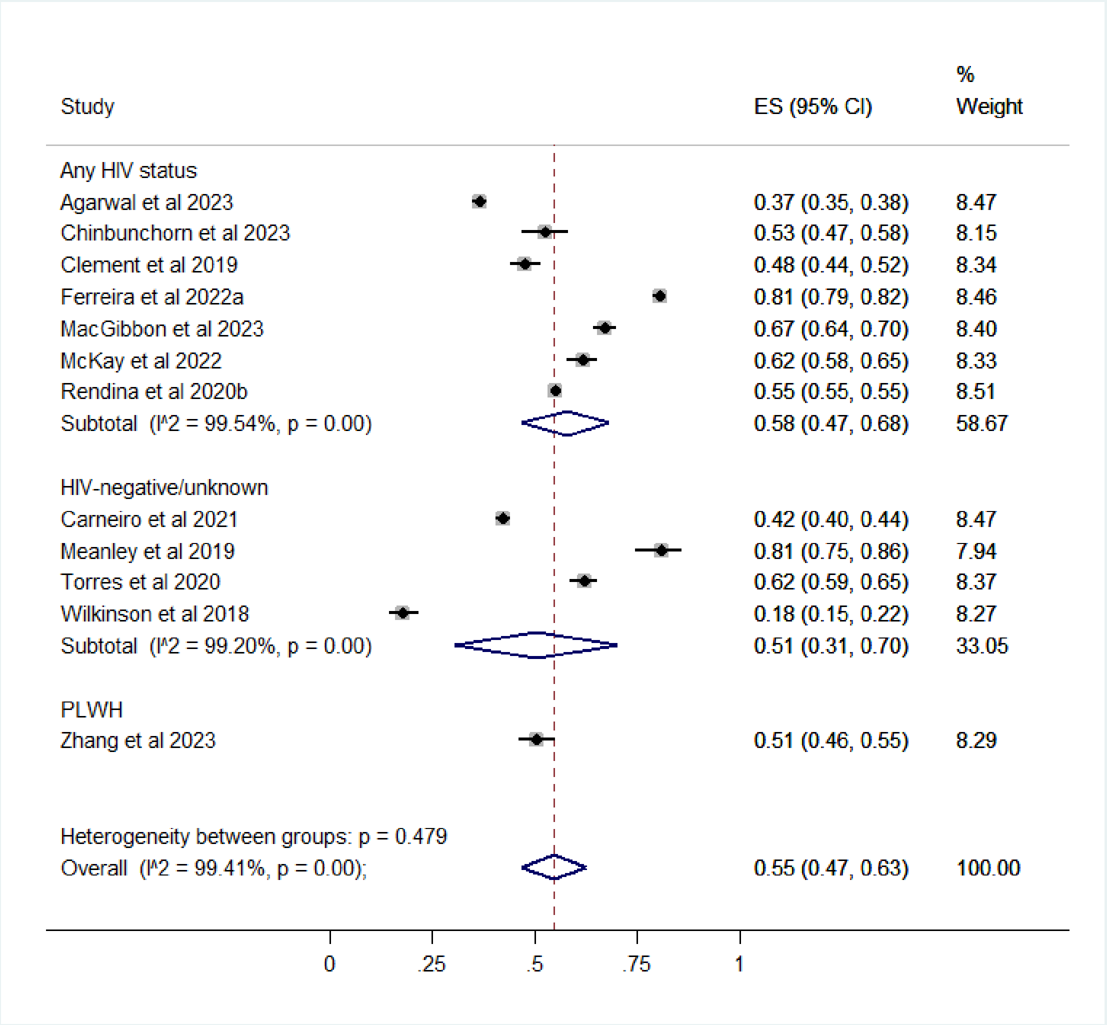

Figure S5: Forest plot describing the prevalence of U=U awareness in PLWH. Subgroup analyses by sexual orientation is presented

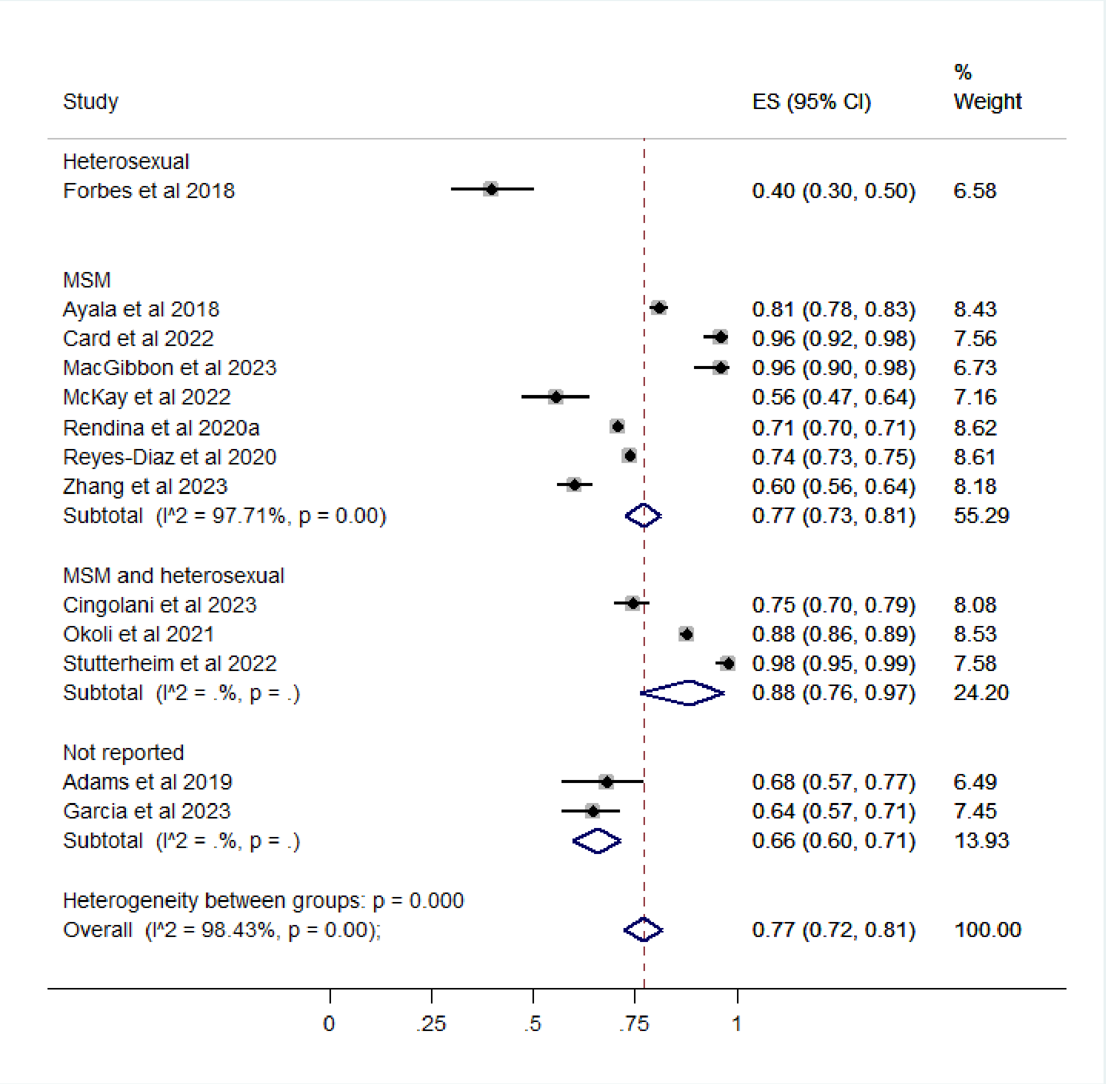

Figure S6: Forest plot describing the prevalence of complete U=U acceptance in PLWH. Subgroup analyses by sexual orientation is presented

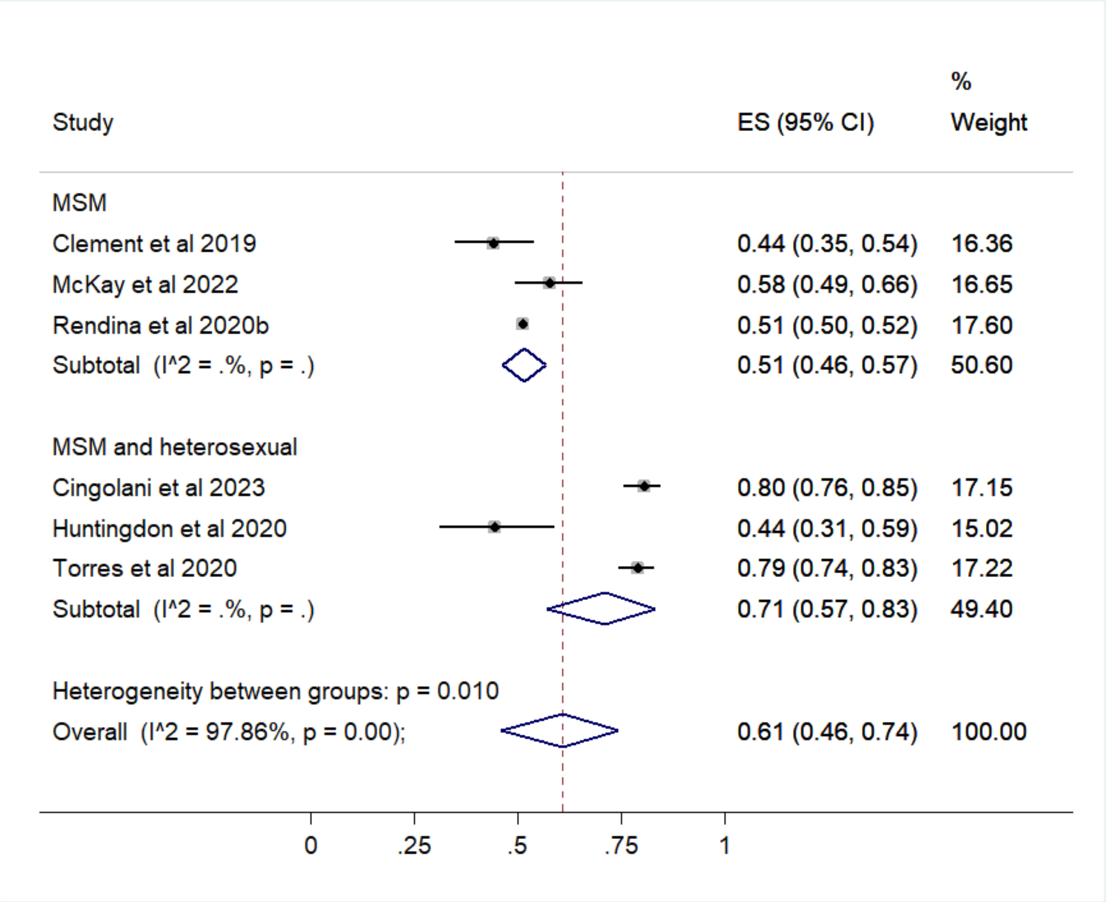

Figure S7: Forest plot describing the prevalence of any U=U acceptance in PLWH. Subgroup analyses by sexual orientation is presented

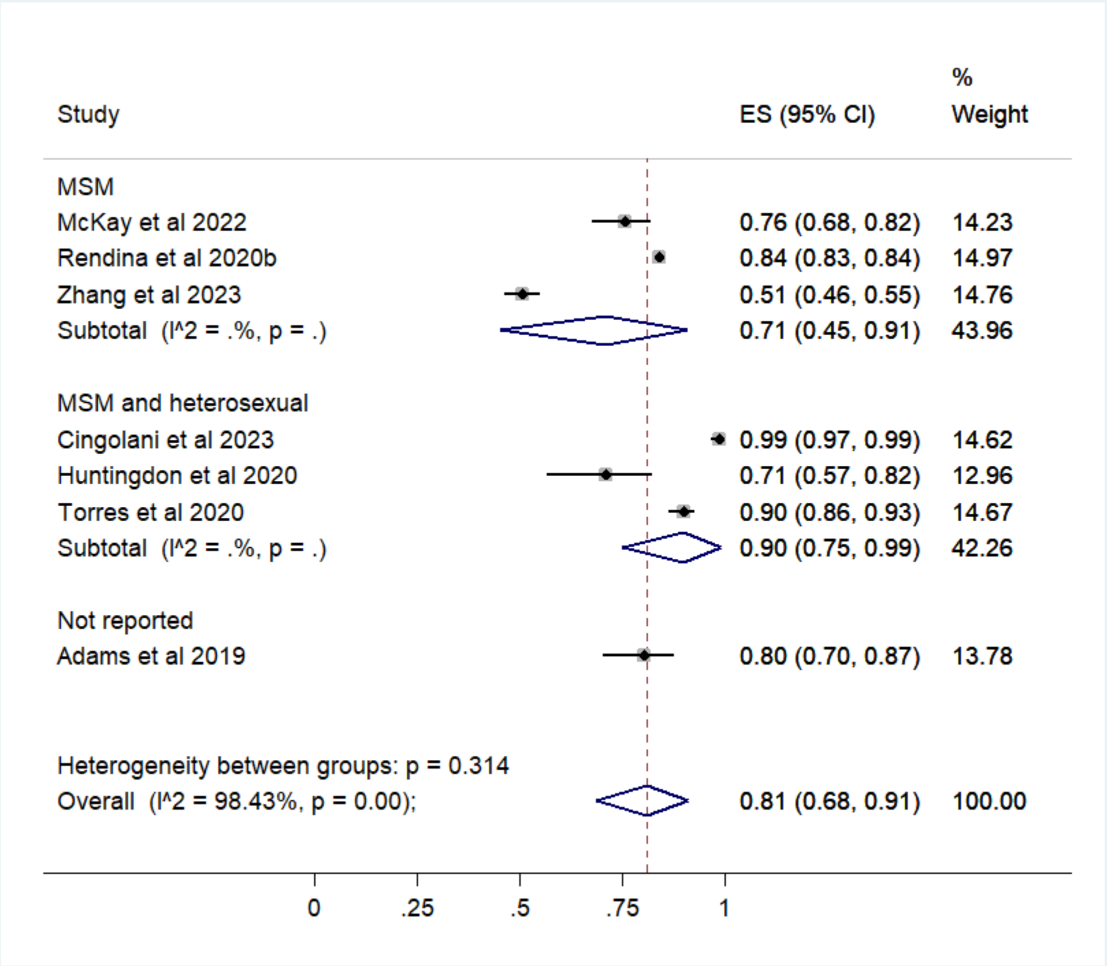

Figure S8. Forest plot describing the prevalence of U=U awareness prevalence in miscellaneous categories.

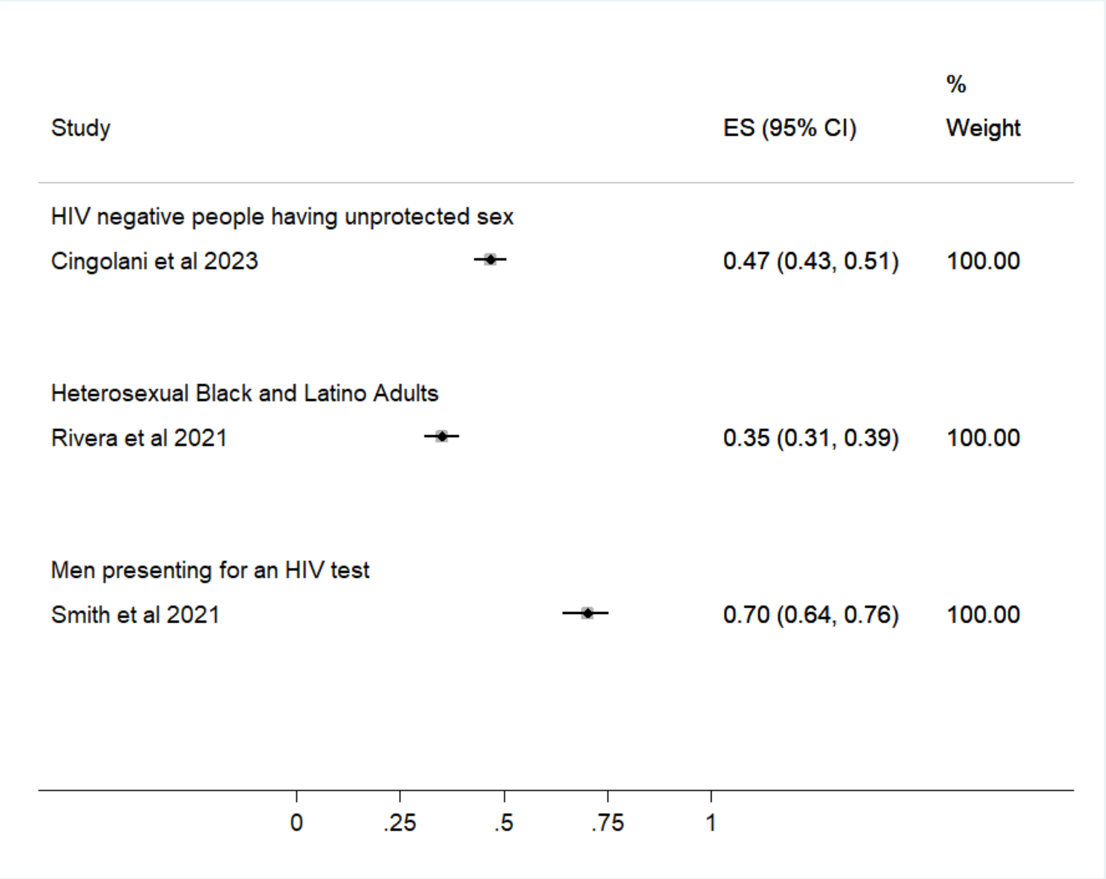

Figure S9. Forest plot describing the prevalence of complete U=U acceptance in miscellaneous categories.

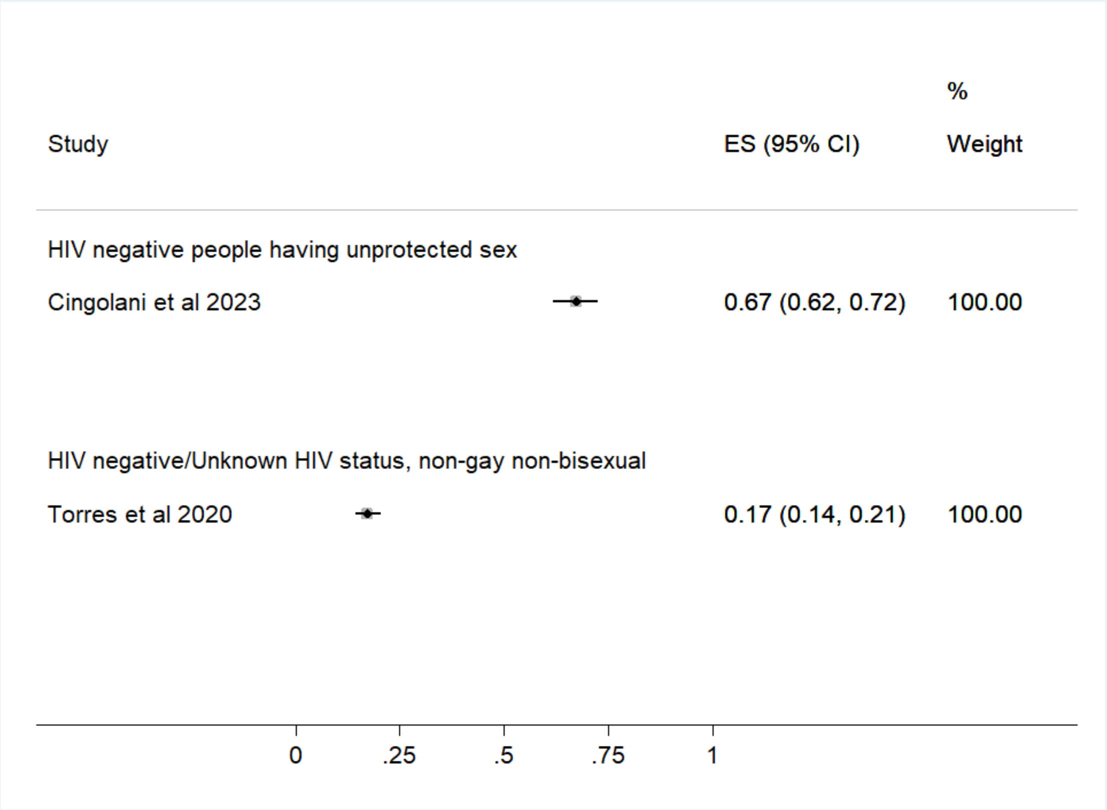

Figure S10. Forest plot describing the prevalence of any U=U acceptance in miscellaneous categories.

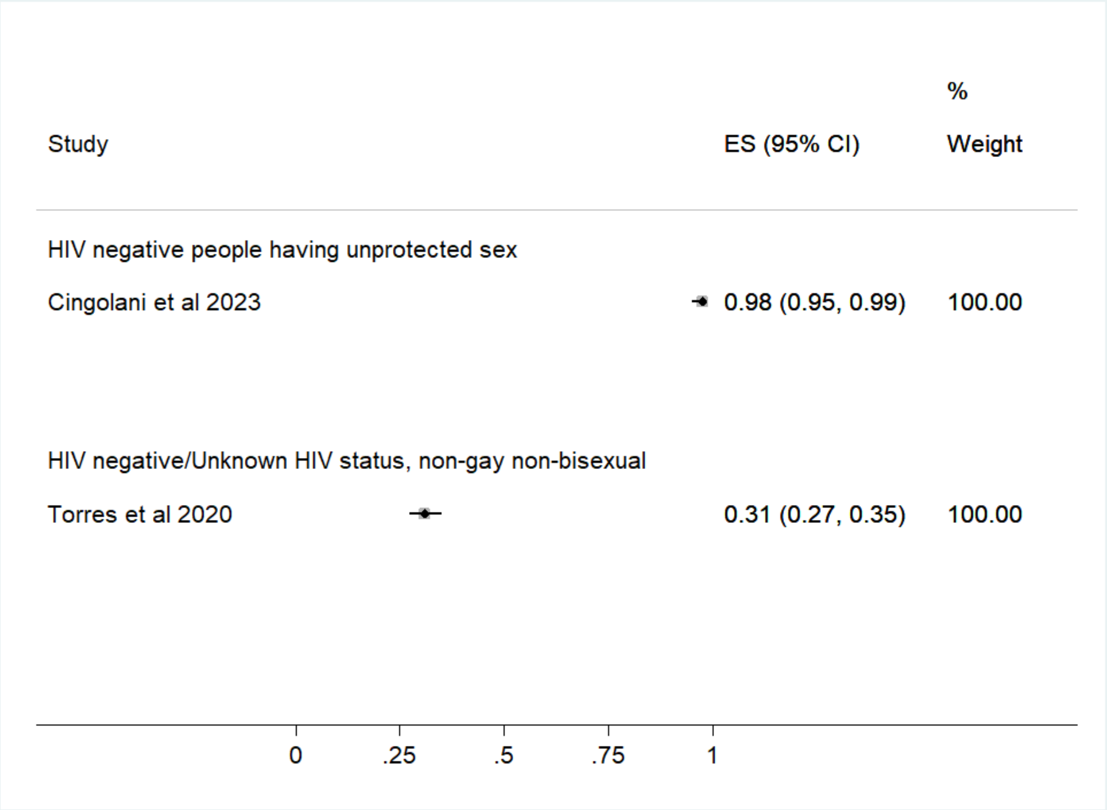

Figure S11. Funnel plot of the meta-analysis on U=U awareness in MSM.

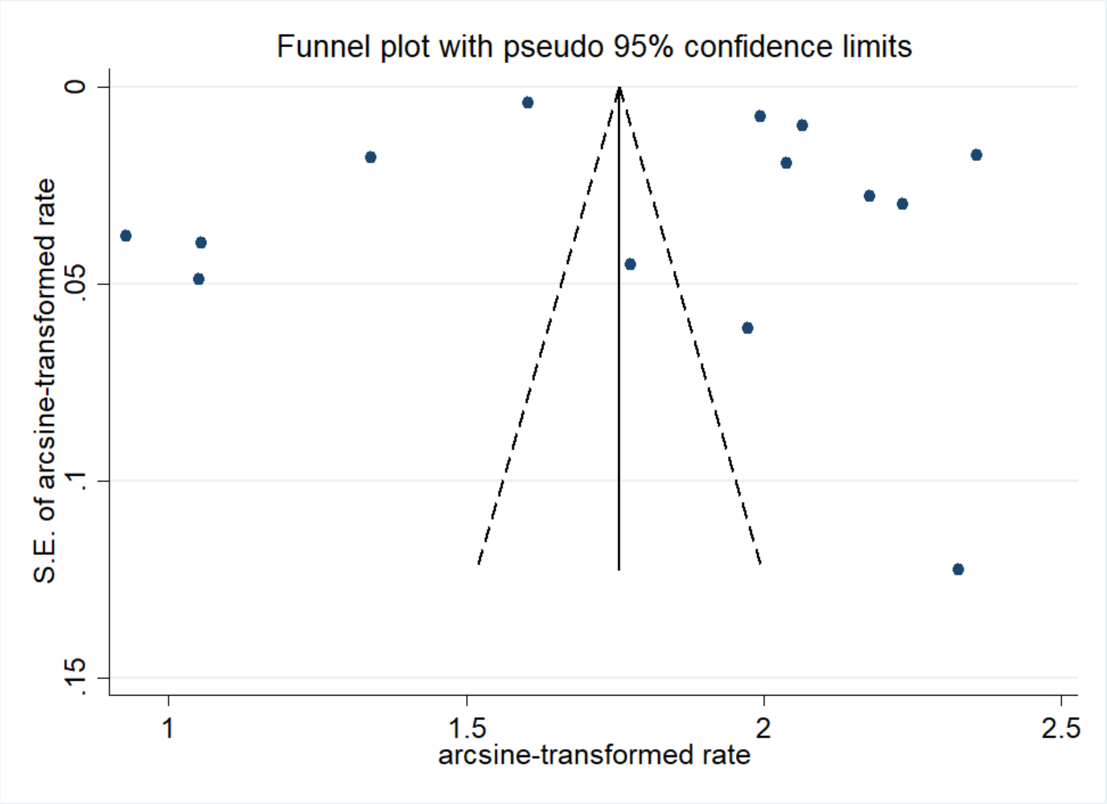

Figure S12. Funnel plot of the meta-analysis on complete U=U acceptance in MSM.

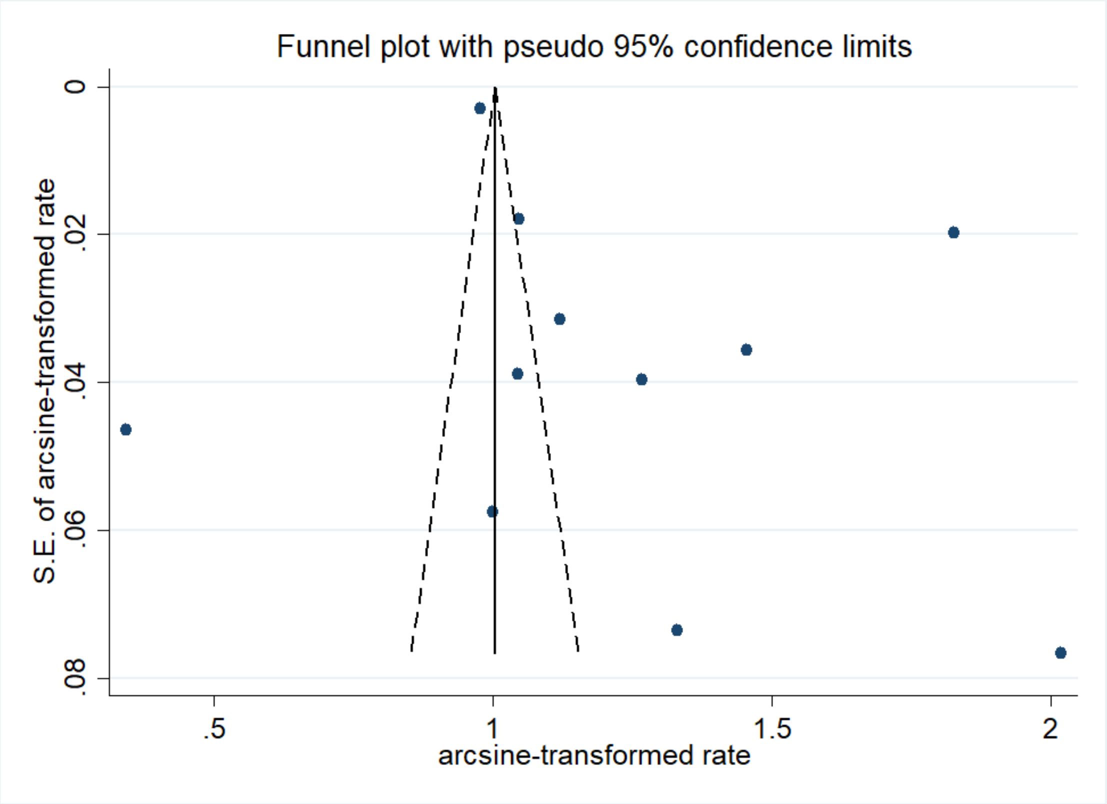

Figure S13. Funnel plot of the meta-analysis on any U=U acceptance in MSM.

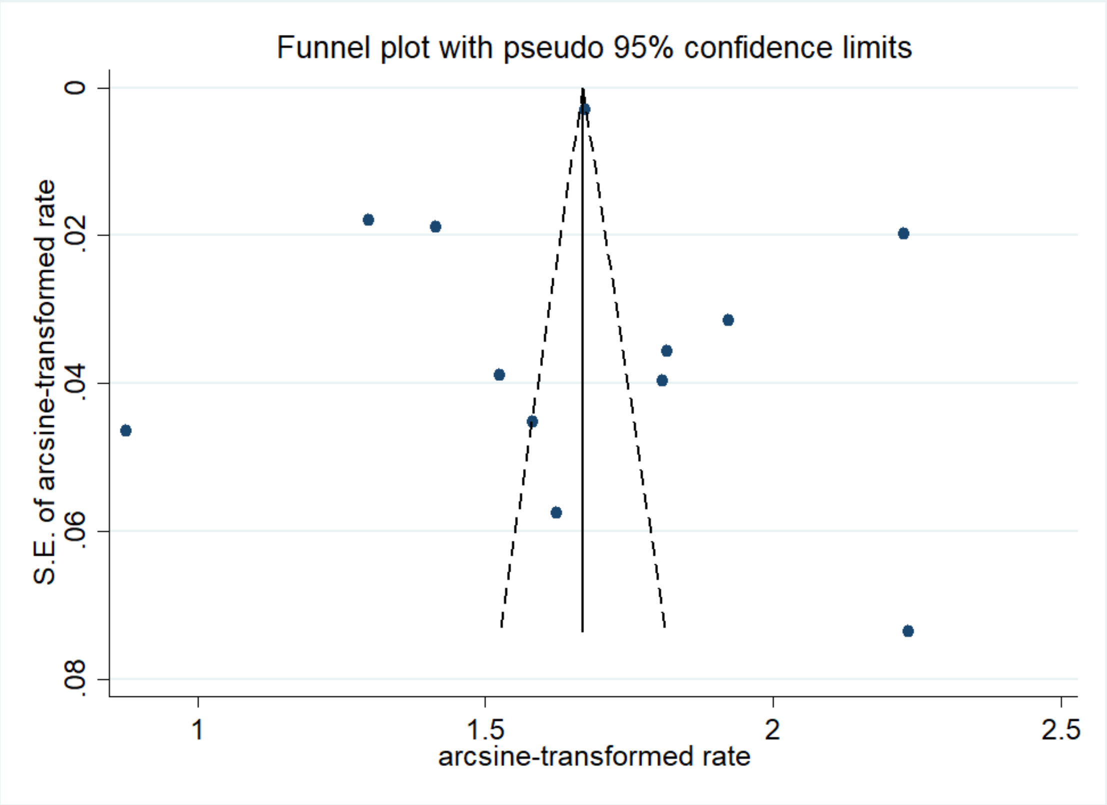

Figure S14. Funnel plot of the meta-analysis on U=U awareness in PLWH.

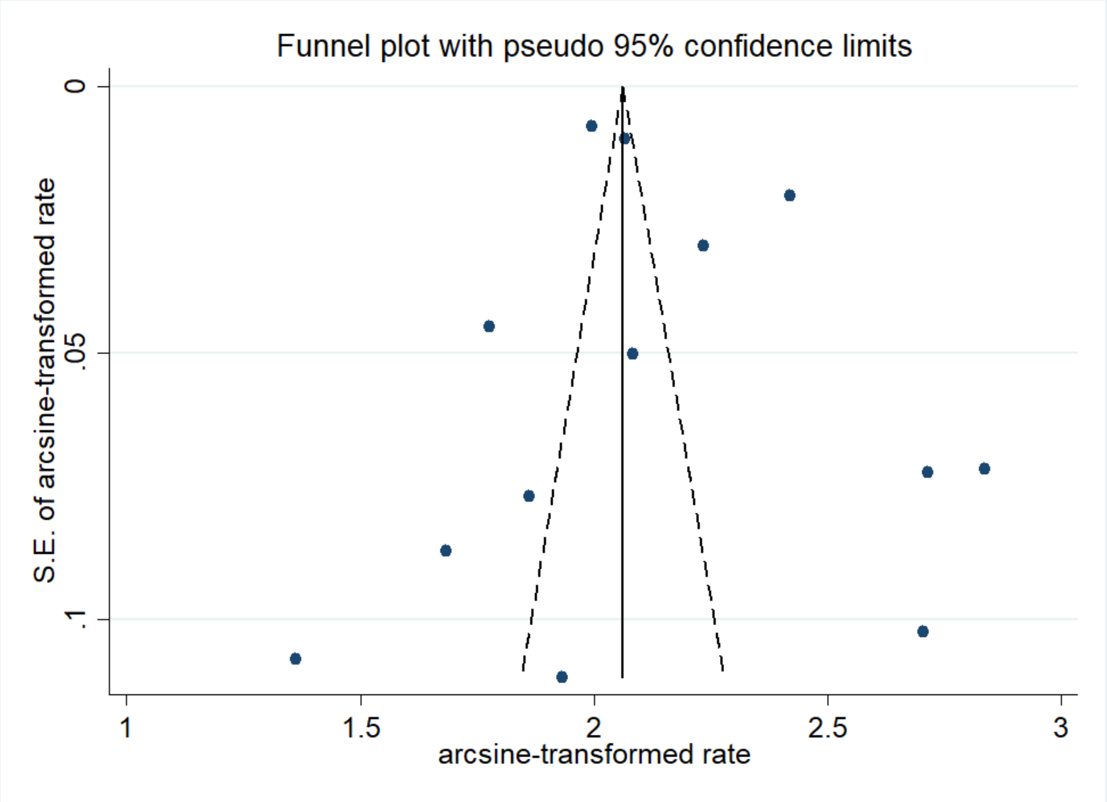

Supplemental Text

PubMed Algorithm

("U=U" OR "U = U" OR "U Equals U" OR "Undetectable = Untransmittable" OR "Undetectable = Untransmittable" OR "Undetectable Equals Untransmittable" OR "Undetectable = Uninfectious" OR "Undetectable = Uninfectious" OR "Undetectable Equals Uninfectious" OR "TasP" OR "TASP" OR "Treatment-as-Prevention" OR "Treatment as Prevention" OR "Undetectable viral load" OR "Untransmittable") AND ("LGBTQ+" OR "MSM" OR "Men who have sex with men" OR "Lesbian" OR "Gay" OR "Bisexual" OR "Transgender" OR "Queer" OR "Questioning" OR "Sexual minority" OR "Gender minority" OR "Sexual orientation" OR "health care" OR "doctor*" OR "nurse*" OR "general population" OR "population" OR "Student*" OR "Youth" OR "Adolescent*") AND ("HIV" OR "Human Immunodeficiency Virus" OR "AIDS" OR "Acquired Immunodeficiency Syndrome") AND ("Acceptability" OR "Stigma" OR "Awareness" OR "Perception" OR "Community attitudes" OR "Public opinion" OR "Patient views" OR "Understanding" OR "Knowledge")

Google Scholar Algorithm

U=U|Undetectible|Untransmittable|TASP|"Treatment as Prevention"
LGBTQ|MSM|Gay|Minority|Doctor|Nurse|Healthcare|Population HIV|AIDS
Acceptability|Stigma|Awareness|Perception|Understanding|Knowledge

Embase Algorithm

Step	Search Queries	Results
1	("U=U" OR "U = U" OR "U Equals U" OR "Undetectable = Untransmittable" OR "Undetectable = Untransmittable" OR "Undetectable Equals Untransmittable" OR "Undetectable = Uninfectious" OR "Undetectable = Uninfectious" OR "Undetectable Equals Uninfectious" OR TasP OR TASP OR "Treatment-as-Prevention" OR "Treatment as Prevention" OR "Undetectable viral load" OR Untransmittable).af	43,967
2	(LGBTQ+ OR MSM OR "Men who have sex with men" OR Lesbian OR Gay OR Bisexual OR Transgender OR Queer OR Questioning OR "Sexual minority" OR "Gender minority" OR "Sexual orientation" OR "health care" OR doctor* OR nurse* OR general population OR population OR Student* OR Youth OR Adolescent*).af	7,833,252
3	(HIV OR "Human Immunodeficiency Virus" OR AIDS OR "Acquired Immunodeficiency Syndrome").af	724,564
4	("Acceptability" OR "Stigma" OR "Awareness" OR "Perception" OR "Community attitudes" OR "Public opinion" OR "Patient views" OR "Understanding" OR "Knowledge").af	3,289,317
5	1 and 2 and 3 and 4	830

References

1 Agaku I, Nkosi L, Gwar JN, Tsafa T. A cross-sectional analysis of U=U as a potential educative Intervention to mitigate HIV stigma among youth living with HIV in South Africa. *Pan Afr Med J* 2022; **41**: 248.

2 Siegel K, Meunier E. Awareness and Perceived Effectiveness of HIV Treatment as Prevention Among Men Who Have Sex with Men in New York City. *AIDS Behav* 2019; **23**: 1974–83.

3 Patterson S, Carter A, Nicholson V, *et al.* Condomless Sex Among Virally Suppressed Women With HIV With Regular HIV-Serodiscordant Sexual Partners in the Era of Treatment as Prevention. *J Acquir Immune Defic Syndr 1999* 2017; **76**: 372–81.

4 Card KG, Armstrong HL, Lachowsky NJ, *et al.* Belief in Treatment as Prevention and Its Relationship to HIV Status and Behavioral Risk. *J Acquir Immune Defic Syndr 1999* 2018; **77**: 8–16.

5 Holt M, Draper BL, Pedrana AE, Wilkinson AL, Stoové M. Comfort Relying on HIV Pre-exposure Prophylaxis and Treatment as Prevention for Condomless Sex: Results of an Online Survey of Australian Gay and Bisexual Men. *AIDS Behav* 2018; **22**: 3617–26.

6 Card KG, Fournier AB, Sorge JT, *et al.* Substance use patterns and awareness of biomedical HIV prevention strategies among sexual and gender minority men in Canada. *AIDS Care* 2020; **32**: 1506–14.

7 Coulibaly K, Bousmah M-A-Q, Ravalihasy A, *et al.* Bridging the knowledge gap of biomedical HIV prevention tools among sub-saharan african immigrants in France. Results from an empowerment-based intervention. *SSM - Popul Health* 2023; **23**: 101468.

8 Couffignal C, Papot E, Etienne A, *et al.* Treatment as prevention (TasP) and perceived sexual changes in behavior among HIV-positive persons: a French survey in infectious diseases departments in Paris. *AIDS Care* 2020; **32**: 811–7.

9 Coulibaly K, Gosselin A, Carillon S, Taeron C, Mbiribindi R, Desgrees Du Lou A. Low knowledge of antiretroviral treatments for the prevention of HIV among precarious immigrants from sub-Saharan Africa living in the greater Paris area: Results from the Makasi project. *PloS One* 2023; **18**: e0287288.

10 Copeland RM, Wilson P, Betancourt G, *et al.* Disparities in HIV knowledge and attitudes toward biomedical interventions among the non-medical HIV workforce in the United States. *AIDS Care* 2017; **29**: 1576–84.

11 Lippman SA, West R, Gómez-Olivé FX, *et al.* Treatment as Prevention-Provider Knowledge and Counseling Lag Behind Global Campaigns. *J Acquir Immune Defic Syndr 1999* 2020; **83**: e9–12.

12 Armstrong HL, Roth EA, Rich A, *et al.* Associations between sexual partner number and HIV risk behaviors: implications for HIV prevention efforts in a Treatment as Prevention (TasP) environment. *AIDS Care* 2018; **30**: 1290–7.

13 Brogan N, Paquette DM, Lachowsky NJ, *et al.* Canadian results from the European Men-who-have-sex-with-men Internet survey (EMIS-2017). *Can Commun Dis Rep Releve Mal Transm Au Can* 2019; **45**: 271–82.

14 Closson K, Chown S, Armstrong HL, *et al.* HIV leadership programming attendance is associated with PrEP and PEP awareness among young, gay, bisexual, and other men who have sex with men in Vancouver, Canada. *BMC Public Health* 2019; **19**: 429.

15 Tairy D, Levy I, Turner D, Livnat Y, Mor Z. Differences in knowledge, attitudes and behaviors of Israeli HIV-uninfected gay men in HIV-discordant vs. concordant steady relationships. *AIDS Care* 2018; **30**: 802–6.

16 Phanuphak N, Ramautarsing R, Chinbunchorn T, *et al.* Implementing a Status-Neutral Approach to HIV in the Asia-Pacific. *Curr HIV/AIDS Rep* 2020; **17**: 422–30.

17 Rendina HJ, Parsons JT. Factors associated with perceived accuracy of the Undetectable = Untransmittable slogan among men who have sex with men: Implications for messaging scale-up and implementation. *J Int AIDS Soc* 2018; **21**. DOI:10.1002/jia2.25055.

18 Cao W, You X, Li J, *et al.* Same-sex behavior disclosure to health care providers associated with greater awareness of pre-exposure prophylaxis. *BMC Public Health* 2021; **21**: 2243.

19 Bond V, Hoddinott G, Viljoen L, Simuyaba M, Musheke M, Seeley J. Good Health and Moral Responsibility: Key Concepts Underlying the Interpretation of Treatment as Prevention in South Africa and Zambia Before Rolling Out Universal HIV Testing and Treatment. *AIDS Patient Care STDs* 2016; **30**: 425–34.

20 Grace D, Daroya E, Gaspar M, *et al.* Gay, bisexual, and queer men's confidence in the Undetectable equals Untransmittable HIV prevention message: longitudinal qualitative analysis of the sexual decision-making of pre-exposure prophylaxis users over time. *Sex Health* 2023; **20**: 223–31.

21 Bavinton BR, Holt M, Grulich AE, Brown G, Zablotska IB, Prestage GP. Willingness to Act upon Beliefs about 'Treatment as Prevention' among Australian Gay and Bisexual Men. *PloS One* 2016; **11**: e0145847.

22 Sharma A, Kahle EM, Sullivan SP, Stephenson R. Birth Cohort Variations Across Functional Knowledge of HIV Prevention Strategies, Perceived Risk, and HIV-Associated Behaviors Among Gay, Bisexual, and Other Men Who Have Sex With Men in the United States. *Am J Mens Health* 2018; **12**: 1824–34.

23 El-Sadr WM, Kurth A, Farrior J, *et al.* Prevention for HIV-infected Persons in HPTN 065: Room for Improvement. https://www.hptn.org/sites/default/files/2016-05/Poster989_Abstract%2016-643_0.pdf.

24 Prati G, Zani B, Pietrantoni L, *et al.* PEP and TasP Awareness among Italian MSM, PLWHA, and High-Risk Heterosexuals and Demographic, Behavioral, and Social Correlates. *PloS One* 2016; **11**: e0157339.

25 Carter A, Lachowsky N, Rich A, *et al.* Gay and bisexual men's awareness and knowledge of treatment as prevention: findings from the Momentum Health Study in Vancouver, Canada. *J Int AIDS Soc* 2015; **18**: 20039.

26 Holt M, Lea T, Murphy DA, *et al.* Australian gay and bisexual men's attitudes to HIV treatment as prevention in repeated, national surveys, 2011-2013. *PloS One* 2014; **9**: e112349.

27 Holt M, Lea T, Schmidt H-M, *et al.* Increasing Belief in the Effectiveness of HIV Treatment as Prevention: Results of Repeated, National Surveys of Australian Gay and Bisexual Men, 2013-15. *AIDS Behav* 2016; **20**: 1564–71.

28 Agarwal H, Yeatts K, Chung SR, Harrison-Quintana J, Torres TS. Perceived Accuracy Around Undetectable = Untransmitable Among Sexual and Gender Minorities Using Smartphones in India. *AIDS Behav* 2023. DOI:10.1007/s10461-023-04212-y.

29 Avelino-Silva VI, Vasconcelos R, Cerqueira NB, Marcus U, Schmidt AJ, Veras MA. Predictors of knowledge of and access to biomedical prevention among MSM and transgender men in Latin America: Results from the Latin American internet survey. *HIV Med* 2022; **23**: 764–73.

30 Ayala G, Santos G-M, Arreola S, Garner A, Makofane K, Howell S. Blue-Ribbon Boys: factors associated with PrEP use, ART use and undetectable viral load among gay app users across six regions of the world. *J Int AIDS Soc* 2018; **21 Suppl 5**: e25130.

31 Cadeliña J. Condom use among men who have sex with men within Metro Manila, Philippines: Associations with attitude towards its use and sexual health outcomes. *Masters Theses* 2019; published online Oct 1. https://animorepository.dlsu.edu.ph/etd_masteral/6614.

32 Cao W, Li J, Sun S, *et al.* HIV Serostatus Disclosure Among Men Who Have Sex with Men in China in the Era of U=U and PrEP. *AIDS Behav* 2022; **26**: 1477–88.

33 Card KG, St Denis F, Higgins R, *et al.* Who knows about U = U? Social positionality and knowledge about the (un)transmissibility of HIV from people with undetectable viral loads. *AIDS Care* 2022; **34**: 753–61.

34 Carneiro PB, Westmoreland DA, Patel VV, Grov C. Awareness and Acceptability of Undetectable = Untransmittable Among a U.S. National Sample of HIV-Negative Sexual and Gender Minorities. *AIDS Behav* 2021; **25**: 634–44.

35 Chinbunchorn T, Thaneerat N, Howell S, *et al.* Assessment of U=U understanding, PrEP awareness, HIV risk behaviours and factors associated with low HIV knowledge among users of Hornet, an online dating application for LGBTQ, in Thailand. *Sex Transm Infect* 2023; **99**: 21–9.

36 Clement ME, Zimmerman R, Grimm J, Schwartz J. 1281. Awareness of U=U Among Gay and Bisexual Men Who Have Sex with Men. *Open Forum Infect Dis* 2019; **6**: S461.

37 Coyne R, Noone C. Investigating the effect of undetectable = untransmittable message frames on HIV stigma: an online experiment. *AIDS Care* 2022; **34**: 55–9.

38 Ferreira RC, Torres TS, Ceccato MDGB, *et al.* Development and Evaluation of Short-Form Measures of the HIV/AIDS Knowledge Assessment Tool Among Sexual and Gender Minorities in Brazil: Cross-sectional Study. *JMIR Public Health Surveill* 2022; **8**: e30676.

39 MacGibbon J, Bavinton BR, Broady TR, *et al.* Familiarity with, perceived accuracy of, and willingness to rely on Undetectable=Untransmittable (U=U) among gay and bisexual men in Australia: results of a national cross-sectional survey. *Sex Health* 2023; **20**: 211–22.

40 McKay T, Akre E-R, Henne J, Kari N, Conway A, Gothelf I. LGBTQ+ Affirming Care May Increase Awareness and Understanding of Undetectable = Untransmittable among Midlife and Older Gay and Bisexual Men in the US South. *Int J Environ Res Public Health* 2022; **19**. DOI:10.3390/ijerph191710534.

41 Meanley S, Connochie D, Bonett S, Flores DD, Bauermeister JA. Awareness and Perceived Accuracy of Undetectable = Untransmittable: A Cross-Sectional Analysis With Implications for Treatment as Prevention Among Young Men Who Have Sex With Men. *Sex Transm Dis* 2019; **46**: 733–6.

42 Meunier E, Siegel K, Sundelson AE, Schrimshaw EW. Stages of Adoption of ‘Treatment as Prevention’ Among HIV-Negative Men Who Have Sex with Men Who Engage in Exchange Sex. *AIDS Patient Care STDs* 2020; **34**: 380–91.

43 Rendina HJ, Talan AJ, Cienfuegos-Szalay J, Carter JA, Shalhav O. Treatment Is More Than Prevention: Perceived Personal and Social Benefits of Undetectable = Untransmittable Messaging Among Sexual Minority Men Living with HIV. *AIDS Patient Care STDs* 2020; **34**: 444–51.

44 Rendina HJ, Cienfuegos-Szalay J, Talan A, Jones SS, Jimenez RH. Growing Acceptability of Undetectable = Untransmittable but Widespread Misunderstanding of Transmission Risk: Findings From a Very Large Sample of Sexual Minority Men in the United States. *J Acquir Immune Defic Syndr 1999* 2020; **83**: 215–22.

45 Reyes-Díaz M, Schmidt A, Veras M, Stuardo V, Casabona J, Cáceres C. IMPACT OF UNDETECTABLE=UNTRANSMISSIBLE (U=U) KNOWLEDGE ON MENTAL HEALTH OUTCOMES AMONG HIV+ MSM IN THE LATIN-AMERICAN MSM INTERNET SURVEY (LAMIS). 2020. https://www.sidastudi.org/es/registro/a53b7fb378a8e080017a1aeacc9b0663.

46 Torres TS, Cox J, Marins LM, *et al.* A call to improve understanding of Undetectable equals Untransmittable (U = U) in Brazil: a web-based survey. *J Int AIDS Soc* 2020; **23**: e25630.

47 Wilkinson AL, Draper BL, Pedrana AE, *et al.* Measuring and understanding the attitudes of Australian gay and bisexual men towards biomedical HIV prevention using cross-sectional data and factor analyses. *Sex Transm Infect* 2018; **94**: 309–14.

48 Zhang KC, Meng XJ, Hu T, *et al.* [Survey on the cognition of the 'undetectable equals untransmittable' concept among HIV-infected men who have sex with men receiving antiviral treatment]. *Zhonghua Liu Xing Bing Xue Za Zhi Zhonghua Liuxingbingxue Zazhi* 2023; **44**: 1610–5.

49 Adams N, Murril J, Rooney F, Waters L. U=U: patient and staff awareness, understanding and impact. 2019. DOI:10.1111/hiv.12738.

50 Cingolani A, Tavelli A, Calvino GV, *et al.* Awareness and perception of accuracy of the Undetectable=Untransmittable message (U=U) in Italy: results from a survey among PLWHA, infectious-diseases physicians and people having unprotected sex. *AIDS Care* 2023; **35**: 923–33.

51 Forbes K. How a sample of the heterosexual HIV+ community in London understand and feel about U=U. HIV Med. 2018;19(Supplement 2):S50. Cited in: Embase at http://ovidsp.ovid.com/ovidweb.cgi?T=JS&PAGE=reference&D=emed19&NEWS=N&AN=621999002. Accessed November 15, 2023. *HIV Med*; **2018;2019**. http://ovidsp.ovid.com/ovidweb.cgi?T=JS&PAGE=reference&D=emed19&NEWS=N&AN=621999002.

52 García ÁL, Martín-Zaragoza L, Rubio-Ruiz L, *et al.* 6ER-031 Knowledge about human immunodeficiency virus (HIV) transmission in people living with HIV in antiretroviral therapy. *Eur J Hosp Pharm* 2023; **30**: A169–A169.

53 Huntingdon B, de Wit J, Duracinsky M, Juraskova I. Belief, Covariates, and Impact of the 'Undetectable = Untransmittable' Message Among People Living with HIV in Australia. *AIDS Patient Care STDs* 2020; **34**: 205–12.

54 Okoli C, Van de Velde N, Richman B, *et al.* Undetectable equals untransmittable (U = U): awareness and associations with health outcomes among people living with HIV in 25 countries. *Sex Transm Infect* 2021; **97**: 18–26.

55 Stutterheim SE, Kuijpers KJR, Walden MI, Finkenflugel RNN, Brokx PAR, Bos AER. Trends in HIV Stigma Experienced by People Living With HIV in the Netherlands: A Comparison of Cross-Sectional Surveys Over Time. *AIDS Educ Prev Off Publ Int Soc AIDS Educ* 2022; **34**: 33–52.

56 Clifford K, Nixon E, Dean G. Developing an educational package for dental staff to address HIV stigma and discrimination. 2021. DOI:10.1111/hiv.13131.

57 Keane A, Heskin J, Lyons F. Service development, education and training. 2020. DOI:10.1111/hiv.12860.

58 Mastrogianni E, Protopapas K, Leonidou L, *et al.* Η έλλειψη βασικών γνώσεων σε επαγγελματίες υγείας αποτελεί βασική αιτία του στίγματος που σχετίζεται με την HIV λοίμωξη - Μελέτη «ΙΑΣΩ». 2021. https://aids.org.gr/wp-content/uploads/2023/02/2021-1125-27_EEMAA_CongressAIDS33_Supplement_D.pdf.

59 Nunes NN, Vasconcelos R, Cortez AL, *et al.* Is U=U consistently known and implemented? A survey among different medical specialists in Brazil. *Int J STD AIDS* 2023; **34**: 395–401.

60 Ripamonti D, Poliseno M, Mazzola G, *et al.* Perceptions of U = U Among Italian Infectious Diseases Specialists: A Nationwide Survey on Providers' Attitudes Toward the Risk of HIV Transmission in Virologically Suppressed Patients. *AIDS Res Hum Retroviruses* 2022; **38**: 847–55.

61 Shongwe M, Mohamed R, Soliman S, *et al.* Healthcare professionals knowledge, attitudes and practices on HIV/AIDS care in our hospitals. 2021. DOI:10.1111/hiv.13183.

62 Wu J, Fairley CK, Grace D, Chow EPF, Ong JJ. Agreement of and discussion with clients about Undetectable equals Untransmissible among general practitioners in Australia: a cross-sectional survey. *Sex Health* 2023; **20**: 242–9.

63 Colpani A, De Vito A, Zauli B, *et al.* How much do we know about HIV and STIs? A comparison between students receiving a brief training and general population. 2021. DOI:10.1111/hiv.13183.

64 Coyne R, Walsh JC, Noone C. Awareness, Understanding and HIV Stigma in Response to Undetectable = Untransmittable Messages: Findings from a Nationally Representative Sample in the United Kingdom. *AIDS Behav* 2022; **26**: 3818–26.

65 De Vito A, Colpani A, Zauli B, *et al.* How Little Do We Know about HIV and STIs Prevention? Results from a Web-Based Survey among the General Population. *Healthc Basel Switz* 2022; **10**. DOI:10.3390/healthcare10061059.

66 Ferreira RC, Torres TS, Marins LMS, Ceccato M das GB, Bezerra DRB, Luz PM. HIV knowledge and its correlation with the Undetectable = Untransmittable slogan in Brazil. *Rev Saude Publica* 2022; **56**: 87.

67 Katsarolis I, Tsami B. Έρευνα κοινής γνώμης για τις γνώσεις, απόψεις και στάσεις για την HIV λοίμωξη στην Ελλάδα. 2021. https://aids.org.gr/wp-content/uploads/2023/02/2021-1125-27_EEMAA_CongressAIDS33_Supplement_D.pdf.

68 Htun WL, Hogson C, Potts S, Carroll K, Sweeeney J. HIV knowledge survey in a district hospital. 2023. DOI:10.1111/hiv.13478.

69 Rivera AV, Carrillo SA, Braunstein SL. Prevalence of U = U Awareness and Its Association with Anticipated HIV Stigma Among Low-Income Heterosexually Active Black and Latino Adults in New York City, 2019. *AIDS Patient Care STDs* 2021; **35**: 370–6.

70 Smith P, Buttenheim A, Schmucker L, Bekker L-G, Thirumurthy H, Joseph Davey D. Undetectable = Untransmittable (U = U) Messaging Increases Uptake of HIV Testing Among Men: Results from a Pilot Cluster Randomized Trial. *AIDS Behav* 2021; **25**. DOI:10.1007/s10461-021-03284-y.
